# *NTP42*, a novel antagonist of the thromboxane receptor, attenuates experimentally induced pulmonary arterial hypertension

**DOI:** 10.1186/s12890-020-1113-2

**Published:** 2020-04-06

**Authors:** Eamon P. Mulvaney, Helen M. Reid, Lucia Bialesova, Annie Bouchard, Dany Salvail, B. Therese Kinsella

**Affiliations:** 1 ATXA Therapeutics Limited, UCD Conway Institute of Biomolecular and Biomedical Research, University College Dublin, Belfield, Dublin 4, Ireland; 2 UCD School of Biomolecular and Biomedical Sciences, UCD Conway Institute of Biomolecular and Biomedical Research, University College Dublin, Belfield, Dublin 4, Ireland; 3 IPS Therapeutique Inc., 3035 Boulevard Industriel, Sherbrooke, QC J1L 2T9 Canada

**Keywords:** Thromboxane, Prostanoid, Thromboxane receptor antagonist, Pulmonary arterial hypertension, PAH, Monocrotaline, *NTP42*

## Abstract

**Background:**

*NTP42* is a novel antagonist of the thromboxane prostanoid receptor (TP), currently in development for the treatment of pulmonary arterial hypertension (PAH). PAH is a devastating disease with multiple pathophysiological hallmarks including excessive pulmonary vasoconstriction, vascular remodelling, inflammation, fibrosis, in situ thrombosis and right ventricular hypertrophy. Signalling through the TP, thromboxane (TX) A_2_ is a potent vasoconstrictor and mediator of platelet aggregation. It is also a pro-mitogenic, pro-inflammatory and pro-fibrotic agent. Moreover, the TP also mediates the adverse actions of the isoprostane 8-iso-prostaglandin F_2α_, a free-radical-derived product of arachidonic acid produced in abundance during oxidative injury. Mechanistically, TP antagonists should treat most of the hallmarks of PAH, including inhibiting the excessive vasoconstriction and pulmonary artery remodelling, in situ thrombosis, inflammation and fibrosis. This study aimed to investigate the efficacy of *NTP42* in the monocrotaline (MCT)-induced PAH rat model, alongside current standard-of-care drugs.

**Methods:**

PAH was induced by subcutaneous injection of 60 mg/kg MCT in male Wistar–Kyoto rats. Animals were assigned into groups: 1. ‘No MCT’; 2. ‘MCT Only’; 3. MCT + *NTP42* (0.25 mg/kg BID); 4. MCT + Sildenafil (50 mg/kg BID), and 5. MCT + Selexipag (1 mg/kg BID), where 28-day drug treatment was initiated within 24 h post-MCT.

**Results:**

From haemodynamic assessments*, NTP42* reduced the MCT-induced PAH, including mean pulmonary arterial pressure (mPAP) and right systolic ventricular pressure (RSVP), being at least comparable to the standard-of-care drugs Sildenafil or Selexipag in bringing about these effects. Moreover, *NTP42* was superior to Sildenafil and Selexipag in significantly reducing pulmonary vascular remodelling, inflammatory mast cell infiltration and fibrosis in MCT-treated animals.

**Conclusions:**

These findings suggest that *NTP42* and antagonism of the TP signalling pathway have a relevant role in alleviating the pathophysiology of PAH, representing a novel therapeutic target with marked benefits over existing standard-of-care therapies.

## Background

Pulmonary arterial hypertension (PAH) is a chronic progressive disorder of the pulmonary vasculature characterised by abnormal remodelling of small, peripheral resistance vessels in the lung. The occlusion of these pulmonary arterioles leads to a persistently elevated pulmonary vascular resistance (PVR), a raised pulmonary arterial pressure (PAP), and ultimately death due to right ventricular hypertrophy, decreased cardiac output and heart failure. Since the recent 2018 World Symposium on Pulmonary Hypertension, PAH is defined haemodynamically by the presence of mean PAP (mPAP) > 20 mmHg alongside an elevated PVR ≥ 3 Wood Units [1]. The underlying pathological hallmarks of PAH include pulmonary arterial endothelial cell (EC) dysfunction, excessive vasoconstriction, pulmonary artery EC and smooth muscle cell (SMC) proliferation, inflammation & fibrosis, in situ thrombosis and right ventricular (RV) hypertrophy.

Currently, PAH is primarily treated with drugs from four major drug classes targeting the endothelin, prostacyclin and nitric oxide (NO) signalling pathways. However, these approved therapies mainly treat the excessive pulmonary vasoconstriction/reduced vasodilation but fail to alter the cardiac and pulmonary remodelling associated with the later phases of the disease. Therefore, there is an urgent unmet medical need for new drugs to new therapeutic targets that offer greater tolerability/compliance and overall efficacy.

An exciting alternative approach to achieving improved vasodilation is to reduce the excessive vasoconstriction by antagonizing/inhibiting the action of the prostanoid thromboxane (TX) A_2_. TXA_2_, synthesised from arachidonic acid (AA) by the sequential actions of cyclooxygenase (COX)-1/COX-2 and TXA_2_ synthase (TXA_2_S), mediates its actions in humans by binding to the TPα and TPβ isoforms of the T Prostanoid receptor (the TP). TXA_2_ is a potent mediator of platelet aggregation and induces constriction of various types of smooth muscle (SM) including vascular, renal and pulmonary SM. In addition, TXA_2_ acts as a potent *pro*-inflammatory and *pro*-mitogenic agent promoting inflammation, fibrosis, blood vessel remodelling and/or restenosis following endothelial injury and is the main COX-derived SM constrictory prostanoid produced within the lung [2–5]. Hence, imbalances in the levels of TXA_2_, or of TXA_2_S or of the TP have been implicated in a range of cardiovascular, renal and pulmonary diseases, including in PAH [2–5]. The TXA_2_-TP axis also regulates key mitogenic/ERK and RhoA-mediated signalling cascades, explaining, at least in part, the role of TXA_2_ in increasing cell proliferation and migration such as occurs in restenosis, vascular remodelling, and in a range of cancers in which the TXA_2_-TP axis is increasingly implicated [2–8].

Moreover, and also critically, the TP not only mediates the actions of TXA_2_ but also those of the isoprostane 8-iso-prostaglandin (PG) F_2α_ (also termed 15-F2t-isoprostane), a non-enzymatic-, free-radical- derived product of arachidonic acid produced in abundance during oxidative injury/hypoxia, including in various cardiovascular and pulmonary diseases [9]. Significantly in the context of PAH, 8-iso-PGF_2α_ is increased over 3-fold in PAH patients and correlates with PAH status and disease progression [2, 9, 10]. A recent study has also demonstrated the pivotal role of 8-iso-PGF_2α_ signalling through the TP in the pathogenesis of pulmonary fibrosis [11], a key hallmark of PAH disease progression. Thus, TP antagonists will have benefits over existing PAH therapies in that they will not only inhibit the adverse actions of TXA_2_ itself but also those of 8-iso-PGF_2α_ which is present in high amounts in the hypoxic environ of the PAH lung [10].

As further evidence of its role in PAH, TXA_2_ or its stable analogues are also used widely to induce pulmonary hypertension (PH) in large preclinical animal models such as the pig [12]. Infusion of the TXA_2_ mimetic U46619 results in stable PH, with significant increases in mPAP, PVR and in cardiac output (CO) [12–14]. Furthermore, within a hypoxia-induced PH model in pigs, disruption of TXA_2_ production and TP signalling attenuates the negative effects on pulmonary and cardiac parameters [15]. Following treatment with the TP antagonist Daltroban, the significant hypoxia-induced rises in mPAP, PVR and mean right atrial pressure were significantly reduced [15].

*NTP42* is a novel antagonist of the TP and is currently in development for the treatment of PAH. During its development, over 250 small chemical compounds were characterised in calcium mobilisation assays in human embryonic kidney (HEK) 293 cells over-expressing TPα and TPβ following stimulation with the TXA_2_ mimetic U46619 or the isoprostane 8-iso-PGF_2α_ [16, 17]. Following this primary screen, prioritised leads were then subject to secondary screening by examining their ability to inhibit TP (U46619)- mediated aggregation of human platelets ex vivo [16, 17]. Key leads in this series, including the drug candidate *NTP42*, were confirmed to display potent antagonist activity, excellent specificity, pharmacokinetic, pharmacodynamic, toxicology and efficacy profiles in a range of in vitro, ex vivo and in vivo pre-clinical models following oral delivery [16, 17]. Mechanistically, TP antagonists such as *NTP42* may be promising therapeutic drugs for PAH, not only inhibiting the excessive vasoconstriction but also preventing the micro-vessel thrombosis and, potentially, limiting the pulmonary artery remodelling, as well as the inflammation and fibrosis found in PAH. In addition, as also stated, TP antagonists will inhibit signalling by 8-iso-PGF_2α_, the free-radical derived isoprostane generated in abundance in the clinical setting of PAH [2, 9–11]. Thus, the aim of this study was to investigate the efficacy of *NTP42* in the monocrotaline (MCT)-induced PAH rat model, alongside current standard-of-care compounds.

## Methods

### Human lung tissue

Lung tissue from patients with PAH and control subjects was obtained from the Royal Papworth Hospital NHS Foundation Trust Tissue Bank (Cambridge, UK). Patients (*n* = 12) had received lung or heart/lung transplantation for PAH and tissue samples were confirmed to display pathology in keeping with PAH. Control lung (*n* = 12) comprised tissue taken from pneumonectomy specimens resected for malignancy, but distant from tumour regions. Written consent was obtained for all tissue samples using Royal Papworth Hospital NHS Foundation Trust Tissue Bank’s ethical approval (East of England - Cambridge East Research Ethics Committee 08/H0304/56 + 5). Haematoxylin and eosin (H&E) and immunohistochemical staining for the TPα and TPβ isoforms of the T Prostanoid receptor (TP) and the I Prostanoid receptor (IP) was subsequently carried out on each section, as detailed in the Supplementary materials.

### Experimental design of preclinical animal studies

All animal studies were performed at the specific-pathogen-free (SPF) facilities of IPS Therapeutique (IPST; Sherbrooke, Quebec, Canada). The institutional animal ethics committee of IPST approved the studies in strict accordance with the guidelines of the Canadian Council on Animal Care and the US NIH Guide for the Care and Use of Laboratory Animals. Male Wistar–Kyoto rats (Charles River Laboratories, St Constant, Quebec, Canada), aged between 8 and 9 weeks and weighing 200–250 g at the time of their enrolment in the studies were randomised according to their body weight into 5 groups (Groups 1–5) spanning two experimental cohorts (Cohorts #1 and #2; see Supplemental Table 1A for composition of animal groups and experimental cohorts). Animals in Groups 2 to 5 received a single dosage of monocrotaline (MCT; 30 mg/ml stock, in DMSO) by subcutaneous injection at 60 mg/kg dosage on the morning of Day 0. Animals in Group 1 received one subcutaneous injection of the MCT vehicle (DMSO; 2 ml/kg) on the morning of Day 0. Drug treatments were initiated on Day 1 and continued until Day 28. During this period, animals were treated twice-daily (BID) by oral gavage (PO) with *NTP42* (0.25 mg/kg BID, Group 3), Sildenafil (50 mg/kg BID, Group 4), Selexipag (0.25 mg/kg BID, Group 5) or, as negative controls, with drug vehicle (0.375% DMSO; Groups 1 and 2). In all cases, drugs/vehicle were delivered in a dosing volume of 2 ml (BID, PO), where drug treatment began within 24 h post-MCT administration. During the treatment period, rats were given food and water ad libitum. The animals were pair-housed for the duration of the study. All animal care and vivarium maintenance were recorded, with documents kept at the test facility. In addition, clinical observations or cage-side parameters were also recorded throughout the study including food and water intake, breathing activity levels, clinical signs of distress, general well-being, etc. and changes in body weight. All procedures were performed under isoflurane-induced, inhalational anaesthesia to minimize suffering. At the end of the study, following haemodynamic evaluations, animals under anaesthesia were euthanised by exsanguination.

### Haemodynamic evaluations

At the end of the treatment period, on the afternoon of Day 28 of treatment, animals were anesthetised with a mixture of 2–2.5% isoflurane (Abbott Laboratories, Montreal, Canada) in 95% O_2_/5% CO_2_, and placed on a heating pad to maintain body temperature. Rats were then tracheotomised and immediately ventilated by means of a positive-pressure rodent respirator set at ~ 10 ml/kg body weight at a frequency of 65–70 strokes/min. A cannula connected to a pressure transducer was inserted into the left femoral artery to measure the systemic arterial blood pressure. Lead II electrocardiogram (ECG) contact electrodes were placed on the rats to continuously monitor the ECG and a pulse oximeter was placed on the left front paw of the animal to measure oxygen saturation. ECG and saturation were monitored continuously during the surgery. The heart was exposed through a sternotomy and a 20G 30 mm Insyte catheter was introduced into the right ventricle (RV) and rapidly hooked up to a saline filled PE-50 catheter connected to a pressure transducer. Following recording of the right ventricular pressure, the catheter was advanced through the pulmonary valve into the pulmonary artery to allow pulmonary pressure recording. Correct positioning of the catheter was achieved by observing clear transitions in diastolic pressures and general pressure waveforms as the catheter transitioned from the ventricle into the artery. Haemodynamic parameters were recorded continuously for the duration of the procedure or until loss of pulmonary arterial pressure signal. Diastolic and systolic pressure values were measured in mmHg using cursors readings in Clampfit 10.2.0.14 module of the pClamp 10.2.0.14 software (Molecular Devices Inc., Foster City, California, USA). Mean systemic or pulmonary arterial pressure values were calculated using the following formula: Mean Pressure = Diastolic Pressure + 1/3 × (Systolic Pressure – Diastolic Pressure). Animal groups were randomized with the aim of scheduling at least one animal per group for each day of terminal surgery.

### Tissue preparation

Following haemodynamic evaluations, the animal was euthanised by exsanguination, the pulmonary circulation was flushed with 0.9% NaCl, and the heart and lungs were removed *en bloc* from the thoracic cavity. The cardiac tissue was dissected to measure the wet weights of the RV and left ventricle including the septum (LV + S) as part of the Fulton’s index for determination of RV hypertrophy. The left lung lobes were fixed in formalin, dissected into blocks from each of the lower, middle and upper regions and processed to formalin-fixed paraffin-embedded (FFPE) tissue blocks.

### Pulmonary histology and vascular morphometry

FFPE tissue blocks from the middle regions of the left lungs from both Cohorts #1 & #2 were subject to sectioning to yield 4 μm sections and stained with H&E to facilitate histological assessment and morphometric analysis to evaluate vascular remodelling. H&E-stained tissue sections were scanned at 40X magnification using the Aperio Slide Scanner ScanScope XT and viewed using QuPath software [18]. Morphometric analysis was subsequently carried out on each section, where measurements for the total and lumen vessel diameter for all arterioles of diameter ≥ 15 μm present in 10 randomly-selected fields/section (10 mm^2^ total tissue area) were recorded, essentially as previously described [19] and as detailed in full in the Supplementary materials. Based on these measurements, values for the lumen:total ratio, medial thickness (μm) and degree of vessel occlusion (%) were calculated. All analyses were carried out in a blinded fashion with overlap by three independent observers.

### Mast cell counting

For assessment of mast cell density, FFPE tissue blocks from the middle regions of the left lungs from Cohort #2 only were sectioned (4 μm) and stained with toluidine blue (0.1% w/v). Toluidine blue-stained tissue sections were scanned at 40X magnification using the Aperio Slide Scanner ScanScope XT. Slides were viewed in QuPath [18], where an annotation was demarcated comprising the entire tissue area, with the major bronchial and vascular regions removed. Toluidine blue-positive mast cells were counted across the entire annotation using the Fiji distribution of NIH ImageJ software [20] and where values are reported as the number of mast cells per mm^2^.

### Pulmonary fibrosis analysis

FFPE tissue blocks from the middle regions of the left lungs from Cohort #2 only were sectioned (4 μm) and stained with Masson’s trichrome connective tissue staining kit, according to the manufacturer’s instructions (ab150686; Abcam). Trichrome-stained tissue sections were scanned at 40X magnification using the Aperio Slide Scanner ScanScope XT. Slides were viewed in QuPath [18], where an annotation was demarcated comprising the entire tissue area, with the major bronchial and vascular regions removed. The percentage fibrotic area was determined in each section using the Fiji distribution of NIH ImageJ software [20] where images were first subjected to colour deconvolution to separate the collagen (blue) staining channel, essentially as previously described [21].

### Statistical analysis

In all cases, potential outliers within each data set were identified based on the method of interquartile range (IQR) with Tukey fences, to exclude values falling outside either; (i) [Quartile 1 (Q1) - 1.5*IQR] (Lower Fence), or, (ii) [Quartile 3 (Q1) + 1.5*IQR] (Upper Fence). Statistical analyses of differences were then carried out using unpaired Student’s t-tests or one-way ANOVA, followed by post-hoc Dunnett’s multiple comparison t-tests where appropriate, employing GraphPad Prism (V6) throughout. All values are expressed as the mean ± standard error of the mean (SEM) and *n* numbers are detailed within the corresponding figure legend. *P* values ≤ 0.05 were considered to indicate statistically significant differences, where *, **, *** and **** denote *P* ≤ 0.05, 0.01, 0.001, and 0.0001, respectively.

## Results

### Expression of the TP in human control and PAH lung tissue

**Fig. 1:**
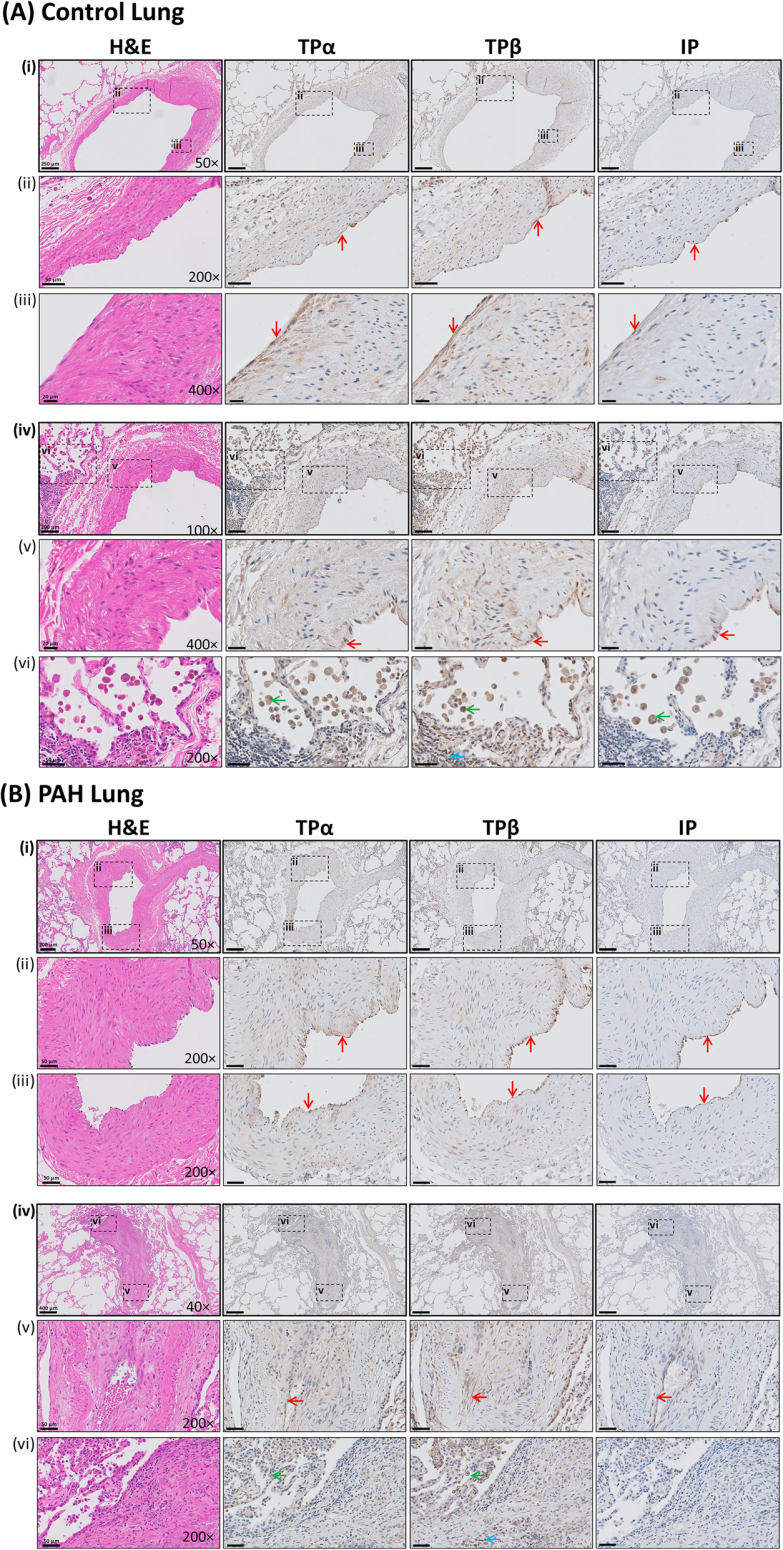
Expression of the IP and TPα/TPβ in control and PAH lung tissue. Representative photomicrographs of haematoxylin and eosin (H&E) and immunohistochemical staining for I Prostanoid (IP) and both TPα and TPβ isoforms of the T Prostanoid receptor (TP), in lungs isolated from (**a**) control individuals and (**b**) PAH patients. In both **Panel A** and **Panel B**, macro images of pulmonary arteries are shown in subpanels (i) and (iv). To enable examination of vascular endothelial and smooth muscle staining, higher magnifications of the pulmonary artery wall, delineated by dotted lines in subpanels (i) and (iv), are displayed in the corresponding subpanels (ii) & (iii) and (v) & (vi), respectively. Within the macro image for each set of panels, black bars representing the respective scale and the magnification of the capture are also displayed. Arrows in the panels highlight example regions referred to in the text. Red arrows highlight vascular endothelium. Blue and green arrows highlight regions of inflammation and alveolar macrophages, respectively

While signalling through the thromboxane receptor (TP) is implicated in the pathogenesis and progression of PAH, few studies have examined the expression of the TP in human PAH lung tissue. Herein, the expression of the TPα and TPβ isoforms of the TP was examined in lung tissue from control individuals and PAH patients (Fig. 1). As a control, expression of the prostacyclin receptor (IP), a related existing PAH drug target, was also investigated.

Positive TPα & TPβ expression was observed in numerous cell types in both control and PAH lung tissue (Fig. 1a & b). Specifically, within the pulmonary arteries, TPα & TPβ were strongly expressed within the vascular endothelium, in both control tissue (Fig. 1a *(ii), 1a (iii) & 1a (v), indicated by arrowheads*) and PAH tissue (Fig. 1b *(ii), 1b (iii) & 1b (v)*). Expression of both TPα & TPβ was also observed within the smooth muscle (SM) of pulmonary arteries, in both control tissue (Fig. 1a *(ii), 1a (iii) & 1a (v)*) and PAH tissue (Fig. 1b *(ii), 1b (iii) & 1b (v)*). Abundant expression of TPα & TPβ was also noted within plexiform lesions, the characteristic morphological hallmark of advanced PAH (Fig. 1b *(iv)*). These complex and disorganised networks of vascular channels result primarily from endothelial hyperproliferation, combined with increased muscularisation, inflammation and fibrosis [22]. Within these structures, TPα & TPβ were expressed in the endothelial core and the surrounding SM (Fig. 1b *(v)*), and also in inflammatory infiltrates within and adjoining the plexiform lesion (Fig. 1b *(vi)*). Furthermore, within immune cells of inflammatory infiltrates in both control and PAH lungs, while predominantly negative for expression of TPα, these infiltrates showed abundant expression of TPβ (Fig. 1a *(vi) & 1b (vi), indicated by arrowheads*). Additionally, strong expression of both TPα and TPβ was noted in alveolar macrophages (Fig. 1a *(vi) & 1b (vi)*). Positive expression of the IP was primarily restricted to the vascular endothelium, being virtually absent from the vascular SM, with staining also evident in alveolar macrophages (Fig. 1a *(ii), 1a (iii) &* 1a *(vi), indicated by arrowheads*).

Hence, these investigations demonstrate abundant expression of both TPα and TPβ isoforms of the TP in the human lung, both in normal control and PAH disease tissues. Bearing in mind this widespread expression of the TP within multiple cell and tissue types within the lung, these findings lend support the investigation of TP antagonism as an approach in the treatment of PAH.

### Effect of *NTP42* on pulmonary and cardiac haemodynamics in the MCT-PAH rat model

A gold-standard preclinical disease model for studying the effects of new drugs on the development of PAH is the monocrotaline (MCT)-induced PAH model, typically carried out in rodents. This study aimed to investigate the efficacy of the novel TP antagonist *NTP42* in an MCT-induced PAH rat model. Throughout these preclinical investigations, the PAH standard-of-care drugs Sildenafil, a cGMP-specific phosphodiesterase type 5 (PDE5) inhibitor, and Selexipag, an oral prostacyclin receptor agonist, were used as reference controls.

**Fig. 2:**
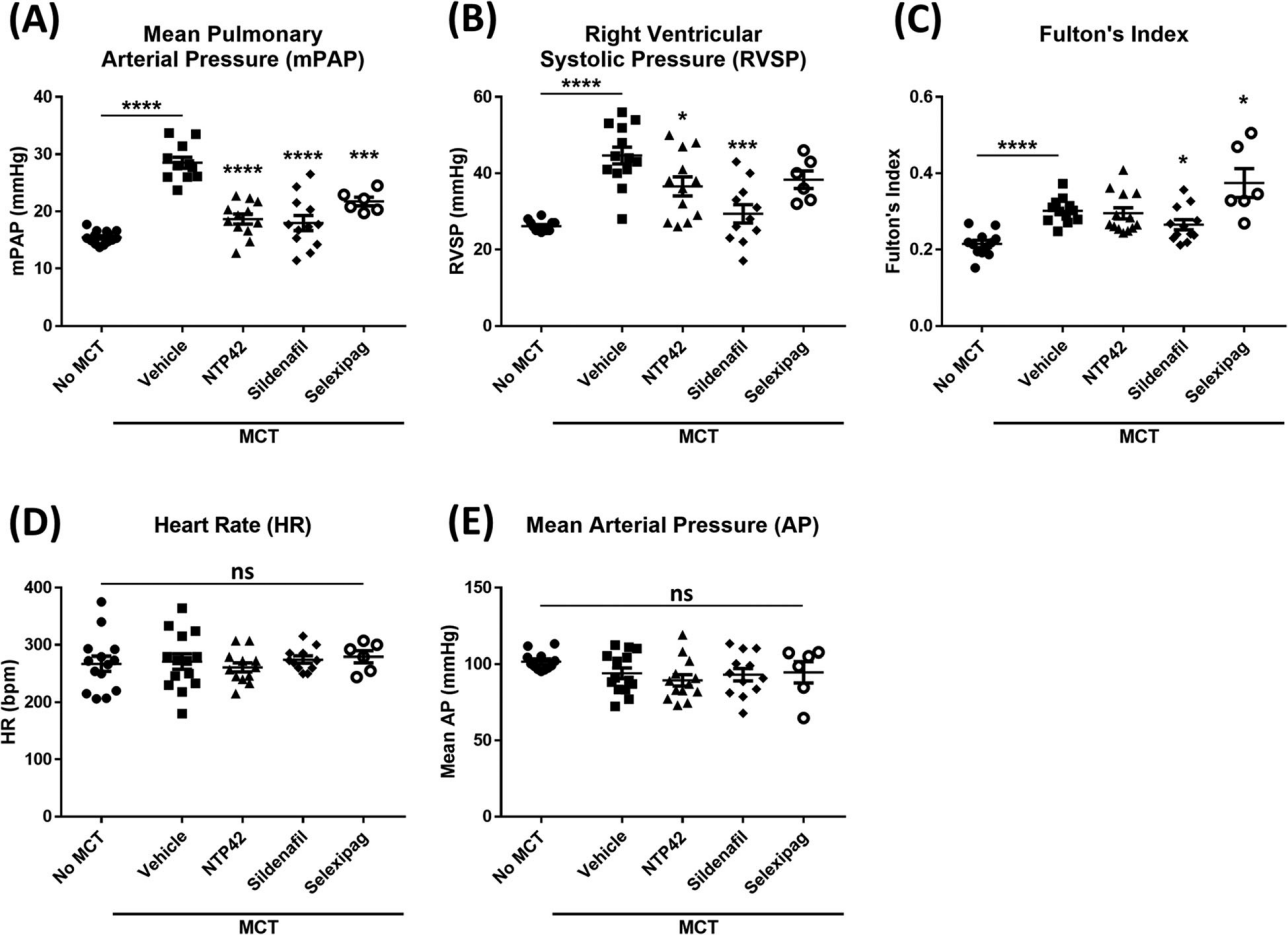
Effect of *NTP42* on haemodynamic parameters in the rat model of MCT-induced PAH. Pulmonary and cardiac haemodynamic measurements of: **a**. The mPAP in the ‘No MCT’, vehicle/‘MCT Only’, *NTP42*, Sildenafil, and Selexipag groups [*n* = 14, 11, 12, 12 and 6, respectively]; **b**. RVSP [*n* = 12, 13, 12, 11 and 6, respectively]; **c**. Fulton’s Index [*n* = 12, 12, 13, 12, and 6, respectively]; **d**. HR [*n* = 14,, 14, 12, 11 and 6, respectively], and (**e**). mAP [*n* = 12, 14, 13, 12 and 6, respectively] are shown for normal control rats (‘No MCT’) and rats treated with MCT (60 mg/kg, s.c) followed by treatment twice daily orally with either drug vehicle (‘MCT Only’ BID, p.o), *NTP42* (0.25 mg/kg BID, p.o.), Sildenafil (50 mg/kg BID, p.o.), or Selexipag (1 mg/kg BID, p.o.) starting from the Day 1 following administration of MCT. All data are expressed as the mean ± SEM. * *P* ≤ 0.05, *** *P* ≤ 0.001, **** *P* ≤ 0.0001 vs. ‘MCT Only’, according to unpaired Student’s t tests (Panels **a**-**c**) or one-way ANOVA (Panels D & E). Note that while Supplemental Table 1A provides details on numbers of animals enrolled into the study and those that survived through to terminal surgery, the numbers (*n*) given in the square brackets in the figure legends refer to the number of input data used for the given experimental parameter following removal of any justifiable outliers identified using the method of interquartile range (IQR) with Tukey fences

The key pulmonary and cardiac haemodynamic findings are presented for each of the treatment groups in Fig. 2. Significant MCT-induced PAH was evidenced by increases in both the mean pulmonary arterial pressure (mPAP; compare 28.5 ± 1.0 mmHg in the ‘MCT Only’-treated group to 15.5 ± 0.3 mmHg for the ‘No MCT’ group, *P* < 0.0001; Fig. 2a) and right ventricular systolic pressure (RVSP; compare 44.7 ± 2.2 mmHg in the ‘MCT Only’-treated group to 26.1 ± 0.4 mmHg for the ‘No MCT’ group, *P* < 0.0001; Fig. 2b) without any significant change observed in either the mean systemic arterial pressure (mAP; Fig. 2d) or heart rate (HR; Fig. 2e). In addition, significant right ventricular remodelling was observed by the increase in the Fulton’s index (right ventricle weight:left ventricle including the septum weight ratio; compare 0.30 ± 0.01 in the ‘MCT Only’-treated group to 0.21 ± 0.01 for the ‘No MCT’ group, *P* < 0.0001; Fig. 2c).

Following treatment with *NTP42*, the MCT-induced increase in the mPAP was significantly reduced (compare 18.7 ± 0.9 mmHg in the *NTP42*-treated group to 28.5 ± 1.0 mmHg for the ‘MCT Only’ group, *P* < 0.0001; Fig. 2a). Similarly, the MCT-induced increase in RVSP was significantly reduced in *NTP42*-treated animals (compare 36.6 ± 2.5 mmHg in the *NTP42*-treated group to 44.7 ± 2.2 mmHg for the ‘MCT Only’ group, *P* = 0.0229; Fig. 2b). There was no significant change observed in the Fulton’s Index following *NTP42* treatment (Fig. 2c). Similar to *NTP42*, both standard-of-care drugs Sildenafil and Selexipag also significantly reduced the MCT-induced increases in mPAP (*P* < 0.0001 and *P* = 0.0003, respectively; Fig. 2a). However, in contrast to both *NTP42* and Sildenafil, treatment with Selexipag did not lead to a significant reduction in the RVSP (*P* = 0.0985; Fig. 2b). Furthermore, while Sildenafil treatment led to a slight improvement in the Fulton’s Index (*P* = 0.0332; Fig. 2c), a significant worsening in this parameter was observed following Selexipag administration (*P* = 0.0224; Fig. 2c).

Taken together, these data demonstrate that *NTP42* reduced the severity of MCT-induced PAH as determined from the haemodynamic measurements of mPAP and RVSP, and at least to a similar or greater extent relative to standard-of-care drugs Sildenafil or Selexipag. Importantly, while treatment with *NTP42* reduced MCT-induced increases in both mPAP and RVSP, it had no deleterious effects on either the systemic mAP or HR (Fig. 2d & e), similar to the standard-of-care drugs tested.

### Effect of *NTP42* on pulmonary vascular remodelling in the MCT-PAH rat model

As stated, vascular remodelling of small pulmonary arterioles is a key feature of PAH, including in the MCT preclinical model of PAH, being a key underlying aetiology that culminates in the elevated pulmonary arterial pressure characteristic of advanced PAH in humans.

**Fig. 3:**
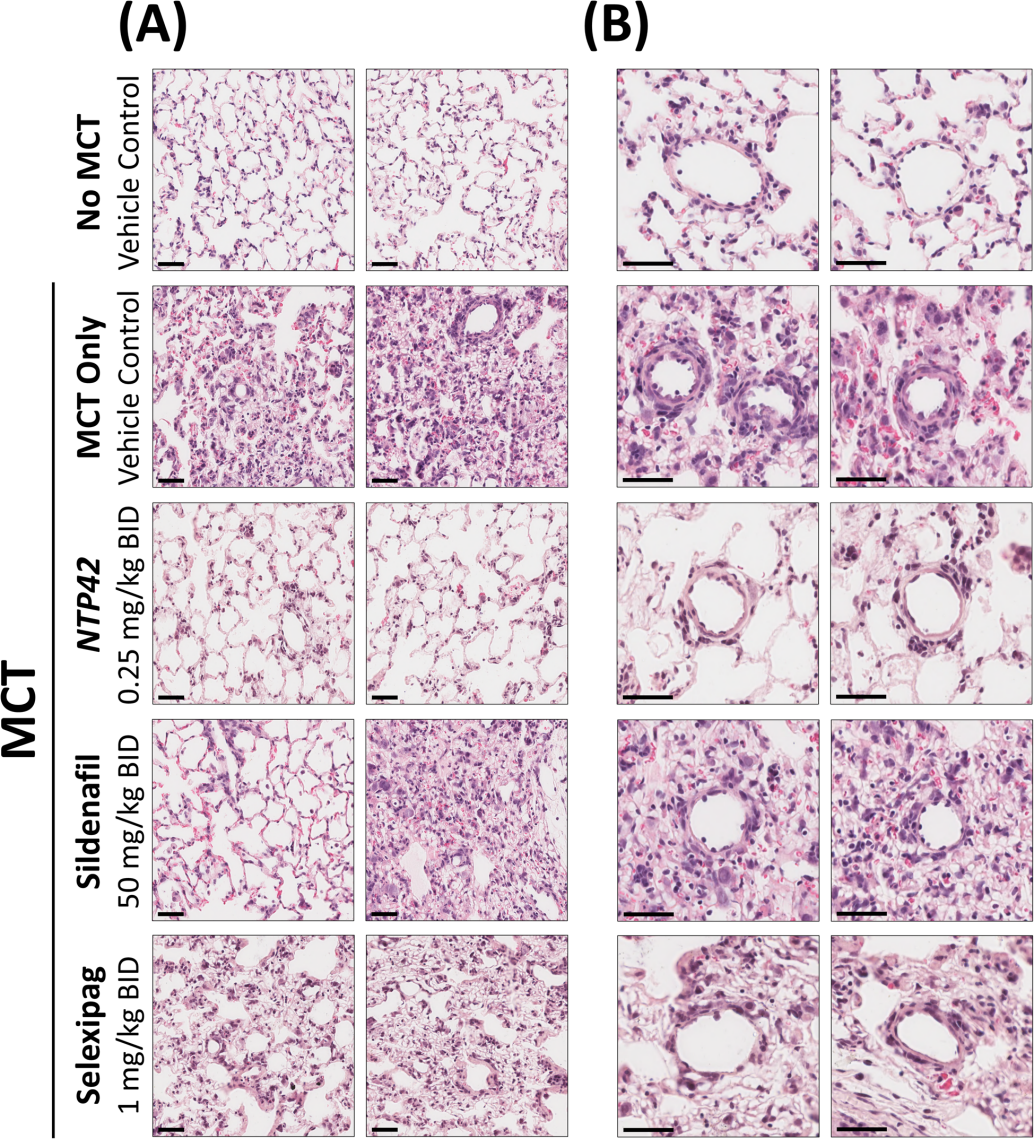
Representative pulmonary histology from the rat model of MCT-induced PAH. Sections from all animals from treatment groups ‘No MCT’, ‘MCT Only’, *NTP42*, Sildenafil and Selexipag were stained with haematoxylin and eosin (H&E) and were digitally scanned using the Aperio ScanScope XT system. **a** Representative photomicrographs of general lung architecture are shown from a randomly selected animal from each treatment group. Images shown were captured at 200× magnification. **b** Representative photomicrographs, with a focus around vascular beds, from a randomly selected animal from each treatment group. Images shown were captured at 400× magnification. Black scale lines represent 50 μm

Representative histology images showing the morphological changes in the lung tissue from the animal groups in the MCT-PAH model, including vascular remodelling of the arterioles, are shown in Fig. 3. In animals within the ‘No MCT’ group, the lung tissue appears generally healthy. Specifically, animals within this group display a loose, open network of lung tissue with normal alveolar wall thickness (Fig. 3a). There is no significant vascular remodelling apparent in the small and medium pulmonary arterioles and there are no apparent sites of appreciable inflammatory infiltration (Fig. 3b). In contrast, animals within the ‘MCT Only’ group present with heavily diseased lung tissue. In these animals, the lung tissue is extremely dense in many regions with heavily thickened gas exchange distances apparent (Fig. 3a). There is a high degree of vascular thickening present, particularly in small and medium vessels, and there are multiple large instances of inflammation and oedema apparent (Fig. 3b).

Similarly, within the Sildenafil- and Selexipag-treated animals, the lung tissue also appears quite diseased (Fig. 3). While the tissues from these animals is generally loose and open, there are multiple dense pockets with heavily thickened gas exchange distances present (Fig. 3a). There are also varying degrees of vascular remodelling apparent, in addition to small pockets of inflammation dispersed throughout the sections from these animals (Fig. 3b). In contrast, lung tissue from *NTP42*-treated animals appeared substantially less diseased (Fig. 3). Reminiscent of findings from the ‘No MCT’- group, *NTP42*-treated animals displayed a loose, open network of lung tissue with normal alveolar wall thickness (Fig. 3a). In these *NTP42*-treated animals, there was a very low degree of vascular remodelling present with only small numbers of sites of substantial inflammatory infiltration present (Fig. 3b).

**Fig. 4:**
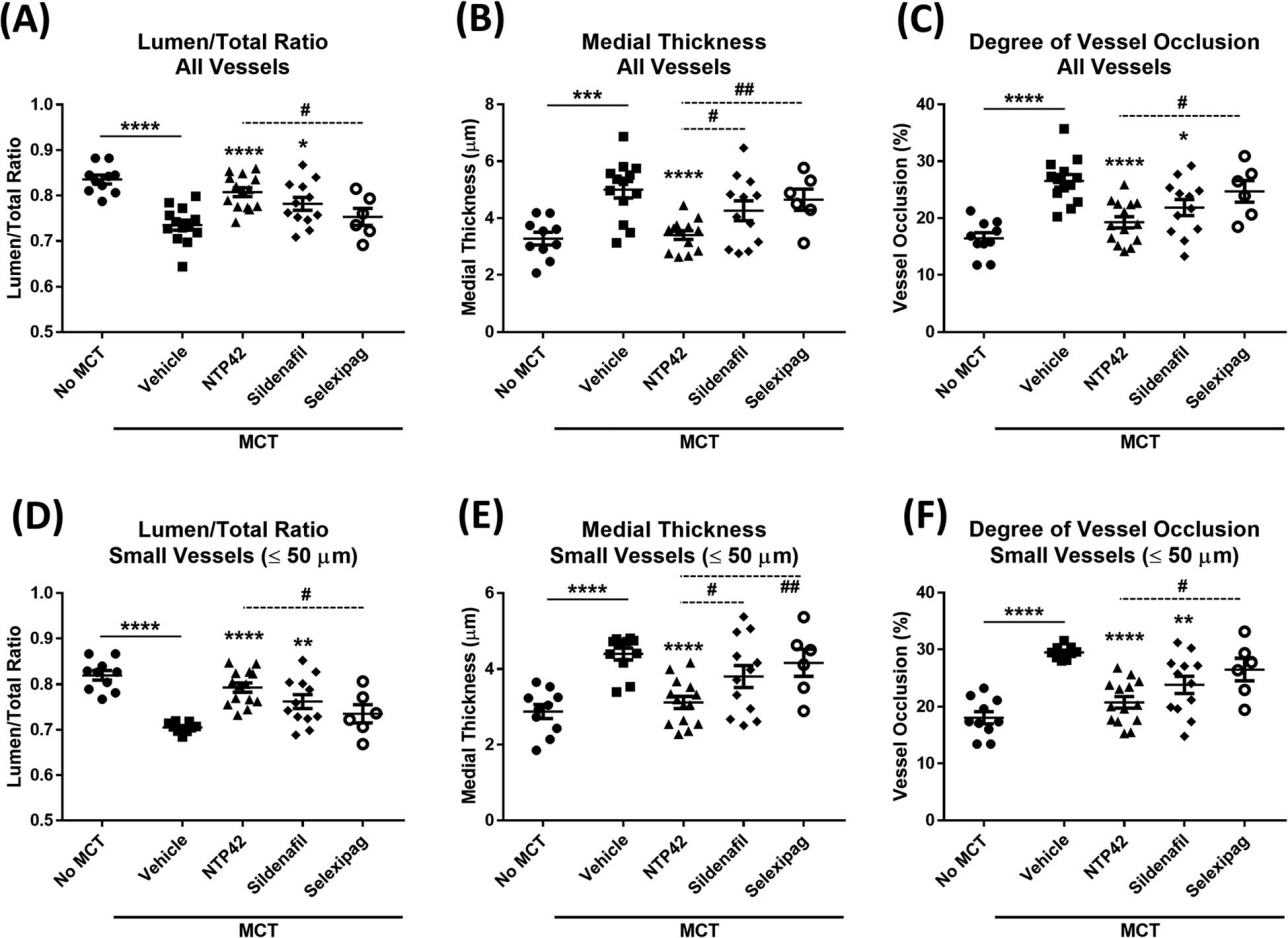
Treatment with *NTP42* attenuates MCT-induced pulmonary vascular remodelling. Detailed morphometric analysis was performed as outlined in the Supplementary Material. **Panels a**-**c** show: (**a**). Lumen:total vessel diameter ratio in the ‘No MCT’, vehicle/‘MCT Only’, *NTP42*, Sildenafil, and Selexipag [*n* = 10, 13, 14, 12 and 6, respectively] in ‘No MCT’, MCT, *NTP42*, Sildenafil, and Selexipag, respectively); (**b**). Medial thickness [*n* = 10, 13, 13, 1 and 6, respectively], and (**c**). Degree of vessel occlusion [*n* = 10, 13, 14, 12 and 6, respectively] within all pulmonary arterioles (≥ 15 μm). **Panels d**-**f**. show: (**d**) Lumen:total vessel diameter ratio [*n* = 10, 9, 14, 12 and 6, respectively]; (**e**). Medial thickness [*n* = 10, 11, 14, 12 and 6, respectively], and (**f**). Degree of vessel occlusion [*n* = 10, 9, 14, 12 and 6, respectively] within small pulmonary arterioles only (≥ 15 μm, ≤ 50 μm). All data are expressed as the mean ± SEM. * *P* ≤ 0.05, ** *P* ≤ 0.01, *** *P* ≤ 0.001, **** *P* ≤ 0.0001 vs. ‘MCT Only’, ^#^ *P* ≤ 0.05, ^##^ *P* ≤ 0.01 vs. *NTP42*, according to unpaired Student’s t tests. Note that the numbers (*n*) given in the square brackets refer to the number of input data used for the given parameter following removal of outliers identified using IQR with Tukey fences

In order to quantify the observed extent of vascular remodelling, detailed morphometric analyses of the pulmonary arterioles in lung tissues was performed across all animals and treatment groups. These morphometric analyses confirmed that MCT treatment led to substantial worsening relative to the ‘No MCT’ animal group in the three parameters reported by this approach, namely, lumen:total vessel diameter ratio, medial thickness, and degree of vessel occlusion (Fig. 4). Notably, this significant vascular remodelling was observed in groupings of either all vessels > 15 μm in size (*P* < 0.0001, *P* = 0.0002 and *P* < 0.0001, respectively; Fig. 4a-c) or in a grouping of small (> 15 μm and ≤ 50 μm) pulmonary arterioles only (all *P* < 0.0001; Fig. 4d-f).

Treatment with *NTP42* significantly attenuated both the MCT-induced decrease in lumen:total vessel diameter ratio and the MCT-induced increase in the degree of vessel occlusion (P < 0.0001, in all cases; Fig. 4a & c). Similarly, treatment with Sildenafil also led to improvements in these parameters relative to the ‘MCT Only’ control (*P* = 0.0152 and *P* = 0.0148, respectively; Fig. 4a & c). Improvements in these pulmonary vascular remodelling parameters were also observed for both *NTP42*- and Sildenafil-treated animals in small vessels only (Fig. 4d & f). Notably, while showing a varying degree of improvement, treatment with Selexipag did not lead to significant changes in either the lumen:total vessel diameter ratio or the degree of vessel occlusion, relative to the ‘MCT Only’ controls (*P* = 0.3976 and *P* = 0.3873, respectively; Fig. 4a & c).

In parallel and consistent with these effects, morphometric analysis also confirmed that the MCT-induced increase in the medial thickness of pulmonary arterioles was significantly reduced in MCT-treated animals following treatment with *NTP42* (*P* < 0.0001), but not following treatment with either Sildenafil or Selexipag (Fig. 4b & e). Moreover, the medial thickness observed following treatment with *NTP42* was significantly reduced relative to both Sildenafil- and Selexipag-treated animals (*P* = 0.0302 and *P* = 0.0018, respectively; Fig. 4b).

Hence, these data strongly support the efficacy of *NTP42* in reducing pulmonary vascular remodelling, and that it is superior to Sildenafil in bringing about these effects, clearly highlighting the potential significant benefits of *NTP42* relative to the standard-of-care reference drugs Sildenafil or Selexipag.

### Effect of *NTP42* on pulmonary inflammation and fibrosis

**Fig. 5:**
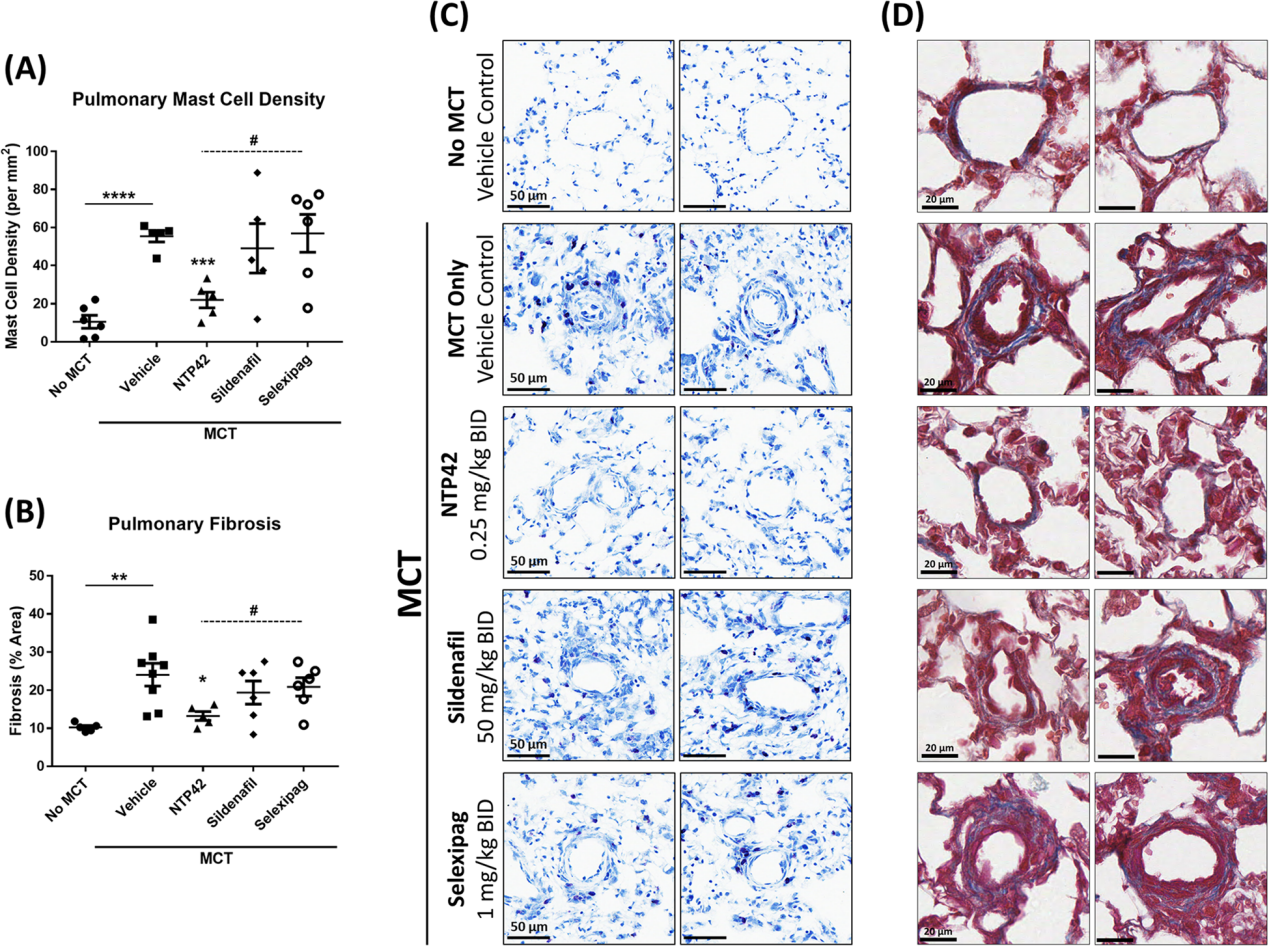
MCT-induced mast cell infiltration and pulmonary fibrosis is attenuated by *NTP42* treatment. Panels **a** & **b** show: (**a**). Pulmonary mast cell density in the ‘No MCT’, vehicle/‘MCT Only’, *NTP42*, Sildenafil, and Selexipag groups [*n* = 6, 5, 5, 5 and 6, respectively] and (**b**). Pulmonary fibrosis [*n* = 5, 8, 5, 6 and 6, respectively]. All data are expressed as the mean ± SEM. * *P* ≤ 0.05, ** *P* ≤ 0.01, *** *P* ≤ 0.001, **** *P* ≤ 0.0001 vs. ‘MCT Only’, ^#^ *P* ≤ 0.05 vs. *NTP42*, according to unpaired Student’s t tests. Panels **c** & **d** show: (**c**). Representative photomicrographs of toluidine blue-stained sections from a randomly selected animal from each group. Images shown were captured at 200× magnification. (**d**). Representative photomicrographs of trichrome-stained sections from a randomly selected animal from each group. Images shown were captured at 400× magnification. Black scale lines represent 50 μm and 20 μm for toluidine blue-stained and Masson's trichrome-stained sections, respectively. Note that the numbers (*n*) given in the square brackets refer to the number of input data used for the given parameter following removal of outliers identified using IQR with Tukey fences

There is growing evidence of the diverse role for inflammatory processes and cell-mediated immunity in the pathogenesis of PAH and indeed other pulmonary conditions [23]. Of the various immune cells that have been implicated in PAH, mast cells were among the first suggested to potentially play a key role in its pathophysiology, strongly contributing to vascular remodelling and leading to pulmonary fibrosis. As such, modulators of mast cells and mast cell-derived mediators may present promising targets for the treatment of lung diseases such as PAH [24]. To evaluate the effect of *NTP42* treatment on mast cell recruitment and the development of pulmonary fibrosis, lung tissues were stained with toluidine blue and Masson’s trichrome to quantity mast cell density and determine collagen deposition, respectively. Results are shown in Fig. 5.

After staining with toluidine blue, lung sections from animals in the ‘MCT Only’ group showed a significantly elevated mast cell density relative to those from the ‘No MCT’ group (P < 0.0001; Fig. 5a). Similarly, after staining with Masson’s trichrome, the percentage fibrotic area in the lungs of animals treated with ‘MCT Only’ was significantly increased compared to those from the ‘No MCT’ group (*P* = 0.0040; Fig. 5b). As displayed in representative photomicrographs in Fig. 5c & d, mast cell recruitment and pulmonary fibrosis in the ‘MCT Only’ group were most pronounced in the immediate vicinity of small pulmonary arterioles.

Treatment with *NTP42* significantly attenuated both the MCT-induced increase in mast cell density and percentage fibrotic area (*P* = 0.0002 and *P* = 0.0180, respectively; Fig. 5a & c). Notably, of the treatments assessed in this study, *NTP42* was the only agent that led to significant and substantial decreases in these parameters. In contrast, treatment with either Sildenafil or Selexipag produced less noticeable changes in the MCT-induced increase in mast cell density and percentage fibrotic area within the lungs of animals treated with these compounds (Fig. 5a & c).

Representative photomicrographs in Fig. 5c & d, with a focus around vascular beds, show a noticeably more pronounced mast cell recruitment and collagen deposition in animals treated with either Sildenafil or Selexipag, compared to those in the *NTP42*-treated group. Furthermore, and in line with the histological assessments and morphometric analyses performed previously (Figs. 3 & 4), pulmonary vascular remodelling in *NTP42*-treated animals appears noticeably reduced relative to the ‘MCT Only’ controls, and indeed relative to the Sildenafil- and Selexipag-treated animal groups.

## Discussion

Treatment of PAH has primarily focused on correcting imbalances between the multiple vasodilator and vasoconstrictor pathways in the pulmonary circulation that results in haemodynamic abnormalities including elevated pulmonary arterial pressure (PAP) and increasing pulmonary vascular resistance (PVR). Unfortunately, currently approved therapies are often short-acting, exhibit limited efficacy at modifying or preventing disease progression, and many have side effects. While the goals of current treatments are to achieve increased exercise capacity, improved quality of life, and to slow disease progression and lower mortality risk, none of the currently-approved therapies have been shown to slow pathological progression [25]. PAH remains an incurable condition with a high mortality rate, underscoring the need for new drugs to new therapeutic targets that will offer greater overall efficacy and tolerability/compliance [26].

TXA_2_, as a potent vasoconstrictor, inducer of platelet aggregation and smooth muscle constrictor and mitogen, has drawn significant attention as a potential therapeutic target for PAH. Several lines of evidence implicate the TXA_2_-TP axis as a contributor to PAH, and indeed in other classes of pulmonary hypertension. The importance of TXA_2_ as a driver of disease pathology is indicated by findings in paediatric patients with PAH caused by congenital heart disease defects. These patients typically have elevated urinary and plasma levels of thromboxane (TX) B_2_, the stable end-product metabolite of TXA_2_ [27]. In infants with persistent pulmonary hypertension of the newborn (PPHN), elevated levels of TXB_2_ positively correlated with a significantly increased pulmonary artery pressures [28]. Furthermore, in adult PAH patients, 24-h excretion levels of TXB_2_ are increased, as compared with normal controls, whereas, in contrast, 24-h excretion of 2,3-dinor-6-keto-prostaglandin F_1α_ (a stable metabolite of prostacyclin) are significantly decreased [29]. Results from a recent randomised clinical trial of Aspirin and Simvastatin (ASA-STAT) showed that PAH patients with higher levels of TXA_2_ were associated with more advanced disease, and with worse clinical outcome/survival [30]. With regards to the TP, employing radioligand binding studies, its expression was shown to be significantly elevated in the right ventricles (RVs) from PAH patients compared to non-diseased controls [31]. Additionally, while expression in normal RV was found to be primarily perinuclear, the TP was strongly expressed throughout the cell surface in cardiomyocytes of patients with PAH [32].

Expanding on the growing evidence for the role for the TP in PAH, this current study demonstrated abundant expression of both TPα and TPβ isoforms of the TP in the human lung, both in normal control and PAH disease tissues. Thereafter, this study demonstrated, for the first time, that TP antagonism by treatment with the novel TP antagonist *NTP42* prevented the development of PAH and ameliorated its progression in the MCT-preclinical model of PAH in rodents. *NTP42* reduced MCT-induced PAH as determined from the haemodynamic measurements, and at least to a similar extent as the standard-of-care drug Sildenafil or Selexipag. Moreover, detailed morphometric analysis of pulmonary vascular remodelling and histological analysis of inflammation and fibrosis indicate that the TP antagonist *NTP42* was superior to Sildenafil and Selexipag in reducing the vascular remodelling, mast cell recruitment and pulmonary collagen deposition in the MCT-treated animals. From a histological assessment, lung tissue from *NTP42*-treated animals and ‘No MCT’ control animals displayed similar histochemistry across the various parameters analysed (i.e., alveolar wall thickening, gas exchange distances, inflammatory infiltrates, vessel remodelling) which, in turn, was radically different and more pronouncedly diseased in ‘MCT Only’-, Sildenafil- and Selexipag-treated animals. These findings illustrate a significant benefit of *NTP42* compared with the approved drugs Sildenafil and Selexipag in lung pathology/histology outcomes. Notably, in the current study, *NTP42* shows at least equivalent haemodynamic outcomes or significantly greater lung histology/pathology benefit than Sildenafil even when *NTP42* (0.25 mg/kg BID, PO) was used at a 200-fold lower dosage than Sildenafil (50 mg/kg BID, PO).

While similar haemodynamic benefits were observed for *NTP42* in comparison to both Sildenafil and Selexipag in alleviating the MCT-induced increase in mPAP, and where *NTP42* and Sildenafil, but not Selexipag, treatment resulted in decreases in RVSP, it was highly notable that TP antagonism via *NTP42* was the only treatment that showed significant decreases in pulmonary mast cell infiltration and fibrosis. Whereas mast cells have traditionally been recognised as critical in allergic and nonallergic immune responses, a growing body of evidence implicates these cells in cardiovascular disease, including in PAH. Mast cell infiltration around small pulmonary vessels has been noted in models of pulmonary hypertension, and indeed within plexiform lesions of PAH patients [33]. They also stimulate the proliferation of endothelial and smooth muscle cells [34, 35]. Through their release of histamine, heparin and chymase as well as multiple other molecules, mast cells contribute strongly to pro-fibrotic activities, either directly through effects on fibroblasts and fibrocytes, or indirectly through the recruitment and activation of various inflammatory and structural cell types [36]. Additionally, and of particular relevance to this study, activated mast cells produce significant quantities of TXA_2_, alongside other prostanoids [37]. Taken together, these previous findings consolidate the hypothesis that mast cells may contribute to the pathogenesis of PAH. The effect of *NTP42* in reducing mast cell recruitment within the lung, and the potential resulting pulmonary fibrosis, is of particular note and demonstrates a unique benefit of TP antagonism in this study, at least. While deemed beyond the scope of the current study, it will be of interest to explore how *NTP42* may impact on other pathways within the innate and/or adaptive immune systems. Furthermore, it must be noted that while toluidine blue is a routinely-used stain for mast cells within the tissues of animals from MCT-PAH investigations [38–40], additional specific markers such as c-Kit/CD117 may be employed in future studies as a further validation of the effect of TP antagonism on mast cell recruitment.

Previous investigations have attempted to clinically assess the effects of disrupting the TXA_2_-TP axis in the treatment of PAH. Inhibition of TXA_2_ production using the TXA_2_ synthase (TXA_2_S) inhibitor CGS13080 resulted in a modest improvement in pulmonary haemodynamics in a small study of patients with PAH [41]. Showing promise in preclinical investigations, the dual TXA_2_S inhibitor/TP antagonist terbogrel prevented pulmonary hypertension and the development of pulmonary artery dysfunction in a chronic hypoxia-induced porcine pulmonary hypertension model [42]. However, a multicentre, randomised, placebo-controlled Phase II trial of terbogrel for use in adults with PAH had to be terminated prematurely because of severe leg pain, which occurred almost exclusively in terbogrel-treated patients [43]. As a TXA_2_S inhibitor, while terbogrel was pharmacologically effective in reducing TXA_2_ metabolites, it also led to a rise in levels of prostacyclin metabolites [43]. In PAH patients, prostacyclin-associated leg pain is a recognised debilitating adverse effect in PAH patients on prostacyclin therapy [44]. In follow-up pharmacokinetic and pharmacodynamic assessments, treatment with terbogrel across a range of doses resulted in up to 10-fold increases in prostacyclin metabolites [45]. Thus, it is now widely accepted that inhibiting TXA_2_ synthesis, either through the administration of selective TXA_2_S inhibitor or dual TXA_2_S inhibitor/TP antagonist compounds, shifts the enzymatic conversion of the common precursor endoperoxide substrates PGG_2_/PGH_2_ away from TXA_2_ biosynthesis towards generation of the pain/nociceptive-inducing prostacyclin [43].

Bearing in mind these side-effects associated with increases in prostacyclin levels as a result of TXA_2_S inhibition, it is crucial that any proposed inhibitor of the TXA_2_-TP signalling axis have a positive drug profile and yet retain the ability to ameliorate the development of PAH. *NTP42* is a highly potent and selective TP antagonist with efficacy in the low nanomolar range (Supplemental Figures 1 & 2). Specifically, *NTP42* antagonised intracellular calcium mobilization following stimulation of mammalian HEK 293 cells stably over-expressing the TPβ isoform of the human TP (HEK.TPβ line) with the TXA_2_ mimetic U46619 (IC_50_ 8.86 ± 3.07 nM; Supplemental Figure 1A). Furthermore, and as previously discussed, as a highly specific antagonist of the TP, *NTP42* will directly inhibit the actions of the isoprostane 8-iso-PGF_2α_, which can exert pulmonary hypertensive effects in several distinct ways [46]. Herein, *NTP42* antagonised intracellular calcium mobilization in the HEK.TPβ line following stimulation with 8-iso-PGF_2α_ (IC_50_ 8.04 ± 3.74 nM; Supplemental Figure 1B). *NTP42* was also shown to antagonize U46619-induced aggregation of human platelets, where *NTP42* was confirmed to have an IC_50_ of 10.6 ± 1.7 nM (Supplemental Figure 2). In the context of TP specificity, *NTP42* was confirmed to be an entirely selective TP antagonist, with no agonist actions at the TP (Supplemental Figure 3). Specifically, *NTP42* was assessed for activity at the 7 other prostanoid receptors, namely the prostaglandin (PG) D_2_ (DP_1_), PGE_2_ (EP_1_, EP_2_, EP_3_, EP_4_), PGF_2α_ (FP) and PGI_2_/prostacyclin (IP) prostanoid receptors, and was confirmed to exhibit no agonist or antagonist effects at these receptors (Supplemental Figure 3 & Supplemental Table 2). Furthermore, unlike the dual TXA_2_S inhibitor/TP antagonist terbogrel, *NTP42* does not inhibit TXA_2_S (Supplemental Figure 4). Thus, in contrast to terbogrel which, as stated, has been clinically evaluated for efficacy in PAH [43], *NTP42* is a selective TP antagonist and does not affect levels of the nociceptive agent prostacyclin.

## Limitations of study

Based on the choice of the PAH preclinical model, several limitations with the current study must be acknowledged. In the case of the MCT-PAH model, animals respond to a single administration of the highly toxic pyrrolizidine alkaloid MCT and the disease develops quickly where even within 6 h post-MCT administration, changes associated with disease induction can be observed. Thereafter, over a period of 1–2 weeks, PAH develops and without any drug intervention, a high proportion of animals typically succumb to the disease through death or animals must be sacrificed due to morbidity concerns [47]. Thus, owing to the relatively rapid development and progression of the disease, the MCT-induced PAH model is frequently best employed as a prophylactic or early intervention model where the test drug is administered either simultaneously with the MCT or within a short duration post-MCT induction, as was the case in this study. The therapeutic effects of *NTP42* should therefore be further investigated using conditions viewed as more reminiscent of a treatment model, rather than a prophylactic model. This could be achieved within the MCT-induced PAH model by delaying treatment with *NTP42* to strike a balance between undue loss of animals due to high mortality rates versus definitively demonstrating treatment of the disease. Alternatively, and in line with recent industry recommendations [48], the therapeutic effects of *NTP42* should be investigated using a second distinct preclinical model, such as the Sugen 5416/hypoxia (SuHx)-induced PAH model. While both the MCT model and the SuHx model are widely reported to emulate PAH, they each display features both typical and atypical of the human disease and of each other [47, 49–51]. For example, a key distinguishing feature between the SuHx and MCT-induced PAH models is the formation of occlusive neointimal or plexiform lesions, which are characteristic of the advanced lesions seen in PAH patients associated with the former, and widely associated with advanced cases of the human condition, but which is absent in the MCT-PAH model [47, 49–51]. On the other hand, inflammation appears to be a key component of the disease that develops in the MCT-model, a feature that is less of a burden in the human PAH lung and that is virtually absent in the SuHx model [47, 49–51].

Notwithstanding the significance of the findings of this study, it should also be acknowledged that the level of the MCT-induced disease, as demonstrated by the low but consistent increase in mPAP, RVSP and Fulton’s Index, was not as severe when compared with previous studies [52, 53]. It is acknowledged that the level of PAH disease induced by MCT administration can vary significantly across species, strains and individual animals [51, 54]. The more moderate PH induced in this model may account for the lack of significant benefit observed for *NTP42* in the Fulton’s Index parameter, and for the lack of benefit observed for Selexipag in the RVSP and Fulton’s Index parameter. Notably, the Selexipag animal group size was lower than the other treatment groups investigated, and this may also have had an effect on the statistical findings. Future preclinical investigations should be performed under more severe disease conditions, to further highlight the potential benefits of *NTP42* or indeed, of the comparator standard-of-care treatments.

A further acknowledged limitation of this study was that while *NTP42* treatment led to reductions in RVSP relative to the ‘MCT Only’ control, no significant changes were observed in RV hypertrophy, as assessed through measurement of the Fulton’s Index. In an additional follow-up study primarily focussing on the effect of *NTP42* on cardiac hypertrophy, *NTP42* treatment at 0.125 mg/kg QD significantly decreased the MCT-induced rise in the Fulton’s Index (Supplemental Figure 5A). In additional assessments of cardiac hypertrophy within this experimental cohort, RV tissues were stained with *anti-*CD31 antibody (Supplemental Figure 5B) which enabled autologous quantitation of both CD31 positive vascular endothelial cells, a measure of vascularization/angiogenesis, and also of cardiomyocyte cross-sectional area, a measure of cardiac hypertrophy. Thus, measurement of these two parameters (i) cardiomyocyte cross-sectional area and (ii) vascularisation per unit area provides a direct assessment of ‘metabolic demand’ and ‘metabolic supply’, respectively, within the RV tissue and when expressed as a ratio, can be used as a measure, so-called Metabolic Index, of ‘Maladaptive hypertrophy’ (where ratio of (i)/(ii) is > 1) or ‘Adaptive hypertrophy’ (where ratio of (i)/(ii) is < 1). In accordance with the Fulton’s Index (Supplemental Figure 5A) findings, treatment with *NTP42* at 0.125 mg/kg QD led to a significant decrease in cardiomyocyte cross-sectional area (Supplemental Figure 5C). While treatment with *NTP42* at this dose had no significant effect on RV vascularization per se (Supplemental Figure 5D), considering both RV hypertrophy and vascularisation together within a “Metabolic Index” parameter, treatment with *NTP42* led to a significant increase in this indicative parameter of cardiac adaptation (Supplemental Figure 5E). Finally, the progress observed in the clinical management of PAH has been largely attributed to the development of combination therapy strategies, and on the staggered escalation of those dual- or even triple-combination therapies based on close monitoring and assessment of clinical outcomes in line with established treatment algorithms [55–57]. Consistent with these clinical guidelines, it will be important to assess the efficacy of *NTP42* alongside other therapies such as PDE5 inhibitors (e.g. Sildenafil), prostacyclin analogues (e.g. Selexipag) or members of the ERA class of PAH treatments.

## Conclusions

**Fig. 6:**
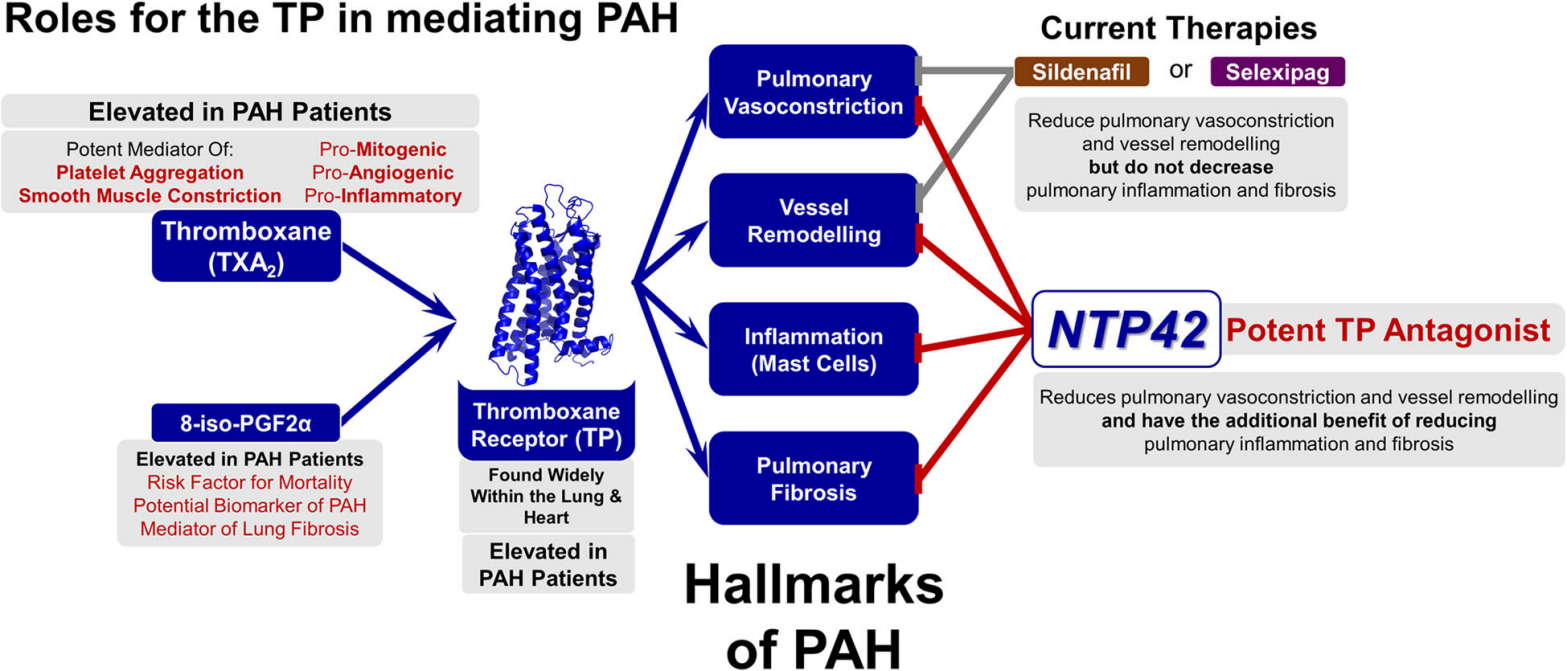
TP antagonism using *NTP42* alleviates key hallmarks associated with PAH. The thromboxane receptor (TP) is a key driver of pulmonary arterial hypertension (PAH). Signalling through the TP, the cyclooxygenase-derived prostanoid thromboxane (TX) A_2_ and free-radical induced isoprostane 8-iso-prostaglandin (PG) F_2α_ both elicit profound effects that contribute to the pathology of PAH, including vasoconstriction and endothelial and smooth muscle cell hyperproliferation, pulmonary vascular remodelling, inflammation, fibrosis, and thrombosis. Within this preclinical study, *NTP42* reduced MCT-induced PAH as determined from the haemodynamic measurements, and at least to a similar extent as the standard-of-care drug Sildenafil or Selexipag. Moreover, *NTP42* was superior to Sildenafil and Selexipag in reducing the vascular remodelling, mast cell recruitment and pulmonary collagen deposition in the MCT-treated animals. Based on these findings, blockade of the TP using *NTP42* is predicted to alleviate the pathophysiology of PAH, representing a novel therapeutic target with marked benefits over existing therapies

In conclusion, and as summarised in Fig. 6, multiple lines of evidence demonstrate the potential importance of TXA_2_ and 8-iso-PGF_2α_, signalling through the TP, as key drivers in the development and progression of PAH, and indeed other classes of pulmonary hypertension. The inhibition of TP signalling by the potent and specific TP antagonist *NTP42* ameliorated the development of PAH over a range of disease-indicating parameters in the MCT-induced preclinical animal model. Uniquely, TP antagonism by *NTP42* led to marked decreases in pulmonary inflammation and fibrosis, hallmarks of PAH pathology which were not alleviated by the other standard-of-care PAH therapies considered within this study. Based on improved efficacy over the spectrum of these disease parameters, *NTP42* is expected to show significantly improved benefits over Sildenafil and Selexipag in the treatment of PAH. Therefore, TP antagonism, such as through the use of *NTP42* is suggested to be a novel therapeutic strategy for the treatment of PAH, with a distinct mechanism of action specifically targeting the pulmonary circulation and alleviating multiple key hallmarks of PAH.

## Supplementary information

**Additional file 1.** Supplementary Methods, Supplementary **Tables 1** & **2** and Supplementary **Figures 1–5**.

## Data Availability

The datasets used and/or analysed during the current study are available from the corresponding author on reasonable request.

## Abbreviations

8-iso-PGF_2α_: 8-iso-prostaglandin F_2α_
AA: Arachidonic acid
CO: Cardiac output
COX: Cyclooxygenase
EC: Endothelial cell
ERA: Endothelin receptor antagonist
FFPE: Formalin-fixed paraffin-embedded
H&E: Haematoxylin and eosin
HR: Heart rate
IP: I prostanoid/prostacyclin receptor
IQR: Interquartile range
mAP: mean systemic arterial pressure
MCT: Monocrotaline
mPAP: mean pulmonary arterial pressure
NO: Nitric oxide
PAH: Pulmonary arterial hypertension
PAP: Pulmonary arterial pressure
PDI: Phosphodiesterase type 5 inhibitor
PVR: Pulmonary vascular resistance
RV: Right ventricle
RVSP: Right systolic ventricular pressure
SEM: Standard error of the mean
sGC: soluble guanylyl cyclase
SM: Smooth muscle
SMC: Smooth muscle cell
SuHx: Sugen 5416/hypoxia
TP: Thromboxane prostanoid receptor
TXA_2_: Thromboxane (TX) A_2_
TXA_2_S: TXA_2_ synthase
