# Supplementary material for: NTP42, a novel antagonist of the thromboxane receptor, attenuates experimentally induced pulmonary arterial hypertension
Source: BMC Pulm Med. 2020 Apr 6;20:85. doi: 10.1186/s12890-020-1113-2 (PMC7132963; doi:10.1186/s12890-020-1113-2)
Supplement: Supplementary file 1 — Additional file 1. Supplementary Methods, Supplementary Tables 1 & 2 and Supplementary Figures 1–5. [file 12890_2020_1113_MOESM1_ESM.docx]

**SUPPLEMENTARY DATA**

***NTP42*, a novel antagonist of the thromboxane receptor, attenuates experimentally induced pulmonary arterial hypertension**

Eamon P. Mulvaney^1^, Helen M. Reid^1, 2^, Lucia Bialesova^1^, Annie Bouchard^3^, Dany Salvail^3^ and B. Therese Kinsella^1, 2^

^1^ ATXA Therapeutics Limited, UCD Conway Institute of Biomolecular and Biomedical Research, University College Dublin, Belfield, Dublin 4, Ireland

^2^ UCD School of Biomolecular and Biomedical Sciences, UCD Conway Institute of Biomolecular and Biomedical Research, University College Dublin, Belfield, Dublin 4, Ireland

^3^ IPS Therapeutique Inc., 3035 Boulevard Industriel, Sherbrooke, QC J1L 2T9, Canada

**Corresponding Author:** Prof. B. Therese Kinsella, PhD, MRIA

ATXA Therapeutics Limited

UCD Conway Institute of Biomolecular and Biomedical Research

University College Dublin

Belfield, Dublin 4

IRELAND

Email: therese.kinsella@ucd.ie; Telephone: +353 1 716 6727

**Supplementary Methods**

**Human lung tissue**

Human lung tissue samples, obtained from the Royal Papworth Hospital NHS Foundation Trust Tissue Bank (Cambridge, UK), were sectioned at 4 μm thickness and baked onto slides at 50–56 °C for 60 min. Sections were dewaxed in two changes of xylene (2 × 10 min incubations) and rehydrated through a series of decreasing alcohol solutions (100%, 3 min × 2; 95%, 1 min; 80%, 1 min) before being washed in double-distilled water (ddH_2_O). Sections were stained with haematoxylin and eosin (H&E) or were subject to immunohistochemical (IHC) staining for the I Prostanoid (IP) and both TPα and TPβ isoforms of the T Prostanoid receptor (TP). For IHC staining, antigen retrieval was first performed by boiling the slides in sodium citrate buffer, pH 6 in a pressure cooker for 5 min. Endogenous peroxidase activity was blocked by incubating the slides in hydrogen peroxide (3% v/v prepared in absolute methanol) for 10 min at room temperature (RT), followed by washing in 1× phosphate-buffered saline (PBS). To block non-specific binding and endogenous biotin, the tissue sections were incubated for 30 min at RT with 5% goat serum in PBS (Blocking Buffer) containing Avidin D (4 drops/ml; Avidin/Biotin Blocking Kit). Sections were then incubated with the desired primary (1°) antibody (~500 μl/slide) for the required incubation period (30 min or overnight) in a humidified chamber, where affinity-purified rabbit *anti*-IP (2.0 μg/ml), *anti*-TPα (9 μg/ml) or *anti*-TPβ (1.8 μg/ml) antibodies were used to detect IP, TPα or TPβ, respectively.(1, 2) All primary antibodies were diluted in Blocking Buffer, containing Biotin (4 drops/ml; Avidin/Biotin Blocking Kit). As a negative control, sections were also incubated with Blocking Buffer in the absence of primary antibody. Following incubation with primary antibody, sections were washed in PBS (3 × 5 min washes; 500 μl/slide) and incubated for 30 min with a biotinylated *anti*-rabbit immunoglobulin (Ig) G secondary antibody prepared in 5% goat serum (1:500). Sections were then washed in PBS (3 × 5 min washes), followed by incubation with streptavidin-conjugated horseradish peroxidase (diluted 1:1500 in Blocking Buffer) for 30 min at room temperature. Thereafter, the slides were incubated with the chromogen 3,3’diaminobenzidine substrate for 5 min. Finally, tissue sections were counterstained for 1 min with haematoxylin (Harris modified), followed by washing in ddH_2_O. Sections were then dehydrated through increasing alcohol series (80%, 1 min; 95%, 1 min; 100%, 3 min × 2) and xylene (2 × 10 min), prior to mounting in DPX. Slides were scanned at 40X magnification using the Aperio Slide Scanner ScanScope XT and viewed using QuPath quantitative pathology and bioimage analysis software(3).

**Pulmonary histology and vascular morphometry**

Formalin-fixed paraffin-embedded (FFPE) tissue blocks from the middle regions of the left lungs from animals within Cohorts #1 & #2 on the monocrotaline (MCT)-induced PAH study (see *Supplemental Table 1*) were subject to sectioning to yield 4 μm thick sections and stained with H&E to facilitate detailed morphometric analysis to evaluate pulmonary vascular remodelling. H&E-stained tissue sections were scanned at 40X magnification using the Aperio Slide Scanner ScanScope XT and viewed using QuPath quantitative pathology and bioimage analysis software(3). The digital image of the tissue section was overlaid with a 1 mm^2^ grid. The squares in the gridded image were numbered where only squares with ≥ 50 % lung tissue present were considered for analysis. Using random number generation in Microsoft Excel, 10 squares were selected and annotated within the image in QuPath, using the grid as a guide to obtain 1 mm^2^ regions for analysis. Note that if any of the squares possessed large or primary blood vessels or significant bronchial tissue, then an adjacent grid square was selected as an alternate. Within each of the squares selected per lung tissue, all blood vessels were measured for luminal and total vessel diameter using the QuPath measurement & annotation tool. Vessels smaller than 15 µm were not included in the analysis. Note that the lumen area was defined as the area within the lamina elastica interna, the layer just below the endothelial cells. Hence, lumen diameter was measured from inner edge of the lamina elastic interna on one side to the inner edge of the other side. For total vessel diameter, measurements were made from the outer edge of the lamina elastic externa on one side to the outer edge of the other side. From these measurements, a lumen:total vessel diameter ratio, medial wall thickness and percentage occlusion may be determined, similar to the method presented previously(4). For vessels that were not presented in cross section or as circular vessels but appeared as longitudinal section or oval shape, lumen and total diameter measurements were taken perpendicular to the mid-point of the longest length of the vessel observed. Lumen and total vessel diameter were appropriately annotated (vessel number, lumen/total) and the table of annotations was exported to Microsoft Excel for analysis. Vessels were categorised as small where the total vessel diameter was ≤ 50 µm. Morphometric analysis was subsequently carried out on each section, where measurements for the total and lumen vessel diameter for all arterioles of diameter ≥ 15 μm present in 10 randomly-selected fields/section (10 mm^2^ total tissue area sampled) were recorded, essentially as previously described(4). The Lumen:Total Ratio was calculated from:  (lumen diameter/total vessel diameter). The medial thickness was calculated from: (total vessel diameter – lumen diameter) / 2. The degree of vessel occlusion was calculated from: [(total vessel diameter – lumen diameter)/total vessel diameter] × 100. All analyses were carried out in a blinded fashion with overlap on a minimum of 20% of all sections by three independent observers.

**Experimental design of preclinical animal studies**

Additional preclinical animal studies (Cohort #3) were also performed to primarily focus on the effect of *NTP42* treatment on cardiac hypertrophy. Experiments were performed at the SPF facilities of IPS Therapeutique (IPST; Sherbrooke, Quebec, Canada). The institutional animal ethics committee of IPST approved the studies in strict accordance with the guidelines of the Canadian Council on Animal Care and the US NIH Guide for the Care and Use of Laboratory Animals. Male Wistar–Kyoto rats (Charles River Laboratories, St Constant, Quebec, Canada), aged between 12-13 weeks at the time of their enrolment in the studies were randomised according to their body weight into 3 groups (Groups 1 – 3; Cohort #3; see *Supplemental Table 1* for composition of animal groups and experimental cohorts). Animals in Groups 2 to 3 received a single dosage of monocrotaline (MCT; 30 mg/ml stock, in DMSO) by subcutaneous injection at 60 mg/kg dosage on the morning of Day 0. Animals in Group 1 received one subcutaneous injection of the MCT vehicle (DMSO; 2 ml/kg) on the morning of Day 0. Drug treatments were initiated on Day 7 and continued until Day 29. During this period, animals were treated once-daily (QD) by oral gavage (PO) with *NTP42* (0.125 mg/kg QD, Group 2) or, as negative control, with drug vehicle (10 % (v/v) DMSO: 10 % (v/v) Cremophor-EL: 80 % (v/v) phosphate-buffered saline (PBS) ; Groups 1 and 2). In all cases, drugs/vehicle were delivered in a dosing volume of 2 ml (QD, PO). During the treatment period, rats were given food and water *ad libitum*. The animals were pair-housed for the duration of the study. All animal care and vivarium maintenance were recorded, with documents kept at the test facility. At the end of the study, animals under anaesthesia (2–2.5% isoflurane (Abbott Laboratories, Montreal, Canada) in 95% O_2_/5% CO_2_) were euthanised by exsanguination. The pulmonary circulation was flushed with 0.9% NaCl, and the heart and lungs were removed en bloc from the thoracic cavity. The cardiac tissue was dissected to measure the wet weights of the RV and LV including the septum (LV+S) as part of the Fulton’s index for determination of right ventricular hypertrophy. The right ventricles were also fixed in formalin and processed to FFPE tissue blocks.

**Cardiac hypertrophy analyses**

FFPE tissue blocks from the RVs from Cohort #3 were subject to sectioning to yield 4 μm sections and were stained with *anti-*CD31 antibody (Abcam; ab182981) and counterstained with haematoxylin to facilitate detailed morphometric analysis to evaluate cardiomyocyte cross-sectional area along with ventricular vascularization. Sections were baked onto slides at 50–56 °C for 60 min, and were dewaxed in two changes of xylene (2 × 10 min incubations) and rehydrated through a series of decreasing alcohol solutions (100%, 3 min × 2; 95%, 1 min; 80%, 1 min) before being washed in double-distilled water (ddH_2_O). For IHC staining, antigen retrieval was first performed by boiling the slides in Tris-EDTA, pH 9.0 in a pressure cooker for 5 min. Endogenous peroxidase activity was blocked by incubating the slides in hydrogen peroxide (3% v/v prepared in absolute methanol) for 10 min at room temperature (RT), followed by washing in 1× phosphate-buffered saline (PBS). To block non-specific binding and endogenous biotin, the tissue sections were incubated for 30 min at RT with 5% goat serum in PBS (Blocking Buffer) containing Avidin D (4 drops/ml; Avidin/Biotin Blocking Kit). Sections were then incubated with the desired primary (1°) antibody (~500 μl/slide) overnight in a humidified chamber, where anti-CD31 (Abcam; ab182981) was used at a dilution of 1:3000. All primary antibodies were diluted in Blocking Buffer, containing Biotin (4 drops/ml; Avidin/Biotin Blocking Kit). As a negative control, sections were also incubated with Blocking Buffer in the absence of primary antibody. Following incubation with primary antibody, sections were washed in PBS (3 × 5 min washes; 500 μl/slide) and incubated for 30 min with a biotinylated *anti*-rabbit immunoglobulin (Ig) G secondary antibody prepared in 5% goat serum (1:500). Sections were then washed in PBS (3 × 5 min washes), followed by incubation with streptavidin-conjugated horseradish peroxidase (diluted 1:1500 in Blocking Buffer) for 30 min at room temperature. Thereafter, the slides were incubated with the chromogen 3,3’diaminobenzidine substrate for 5 min. Finally, tissue sections were counterstained for 1 min with haematoxylin (Harris modified), followed by washing in ddH_2_O. Sections were then dehydrated through increasing alcohol series (80%, 1 min; 95%, 1 min; 100%, 3 min × 2) and xylene (2 × 10 min), prior to mounting in DPX. CD31-stained tissue sections were scanned at 40X magnification using the Aperio Slide Scanner ScanScope XT and viewed using QuPath software(3). Morphometric analysis of cardiomyocyte cross-sectional area was carried out on each of the *anti-*CD31-stained RV sections, where measurements for the total area for all cardiomyocytes present in 8 randomly-selected fields/section (8 x 0.0225 mm^2^) were recorded. Morphometric analysis of ventricular vascularization was also carried out on each of the *anti-*CD31-stained RV sections, where measurements for the total number of CD31^+^ capillaries present in 15 fields/section (15 x 0.0225 mm^2^) were recorded. Only fields where the RV section contained cardiomyocytes predominantly cut in cross-section, rather than longitudinally, were included in the analysis. Based on these measurements, values for the mean Cardiomyocyte Size (μm^2^) and the mean Right Ventricular Vascularization (Capillaries/mm^2^) were calculated. Based on these two measurements, values for the mean ‘Metabolic Index’ were calculated from Ventricular Vascularization (Capillaries/mm^2^) divided by Cardiomyocyte Cross-sectional Area (μm^2^), where in each case the resulting value was normalized to the mean of the No MCT vehicle control group from the particular experimental cohort.

**Measurement of intracellular calcium ([Ca^2+^]_i_) mobilisation**

The human embryonic kidney (HEK) 293 cell line stably over-expressing a hemagglutinin (HA) epitope-tagged form of the TPβ isoform of the human TP (HEK.TPβ) has been previously described.(5) HEK.TPβ cells were routinely cultured in minimal essential medium (MEM) with Earle's salts supplemented with 10% foetal bovine serum (FBS) and maintained at 37 °C in 5 % CO_2_. Prior to performing calcium mobilisation assays, HEK.TPβ cells were washed and harvested in Krebs-HEPES buffer (118 mM NaCl, 4.7 mM KCl, 1.2 mM MgSO_4_, 1.2 mM KH_2_PO_4_, 4.2 mM NaHCO_3_, 11.7 mM D-glucose, 1.3 mM CaCl_2_, 10 mM HEPES, pH 7.4). Cells were then incubated with 3 μM Fluo-4 AM (ThermoFisher Scientific) in Krebs-HEPES buffer containing 1% Pluronic F-127 for 1 hour at 25 °C. The cells were washed and diluted with Krebs-HEPES buffer containing 0.5 % bovine serum albumin (BSA), to provide a final concentration of 3 x 10^5^ cells/ml. Cells were then plated in black 96-well microplates at a density of approximately 50,000 cells/well (160 μl) with either vehicle (0.1 % DMSO) or with the TP antagonist, *NTP42*, where the antagonist concentration was 10X the desired concentration (0.00001 - 10 µM) in 20 μl volume. Fluorescence measurements were performed using the Fluoroskan Ascent microplate fluorometer (ThermoFisher Scientific) based on the protocol described previously.(6) In brief, fluorescence intensity was measured at 520 nm emission wavelength (excitation wavelength 485 nm) for 20 seconds to monitor baseline prior to the addition (20 μl) of the agonists U46619 or 8-iso-PGF_2α_, to achieve the final concentration of 1 μM or 10 μM, respectively. Fluorescence intensity was monitored for a further 120 seconds. Calibration of the fluorescence intensity was performed in each sample by adding 1% Triton X-100 to obtain the maximal fluorescence ratio (F_max_) and then 300 mM EGTA to obtain the minimal fluorescence ratio (F_min_). Intracellular Ca^2+^ ([Ca^2+^]_i_) mobilisation was then calculated from: [Ca^2+^] = Kd(F - F_min_)/(F_max_ - F), where the equilibrium dissociation constant (Kd) for Fluo-4 AM is 345 nM.

**Measurement of platelet aggregation**

Approximately 50 ml of blood was obtained through venepuncture from healthy volunteers, who had not taken medication for 14 days, into syringes containing indomethacin (10 µM) and 3.8% sodium citrate (9:1 v/v) (final concentration, 0.38% sodium citrate). Blood was centrifuged for 10 minutes at 160 × g to obtain platelet rich plasma (PRP). Platelet poor plasma (PPP) was prepared by centrifuging the remaining blood for 15 minutes centrifugation at 900 × g. Platelet aggregation was assessed by light transmittance aggregometry using a PAP-8E platelet aggregation profiler (Bio/Data Corporation). Aliquots of PRP (300 µl) were pre-incubated for 10 min with *NTP42*, where 2-fold serial dilutions from 250 – 3.8 nM were prepared for each, prior to stimulating platelets with 1 µM U46619, incubated at 37 °C, with stirring. The results are presented as the mean (± S.E.M.) Percentage Aggregation determined by changes in light transmission over time as a function of the log concentration of *NTP42*.

**Supplementary Tables**

***Supplemental Table 1***

***Supplemental Table 1A***

| **Group**  **#** | **Group Description** | **Treatment Dose** | ***Cohort #1 (N)*** | | ***Cohort #2 (N)*** | | **Total^#^** |
| --- | --- | --- | --- | --- | --- | --- | --- |
|  |  |  | **Enrolled** | **Deaths*** | **Enrolled** | **Deaths*** |  |
| 1 | No MCT (Vehicle) | BID | 8 | 0 | 6 | 0 | 14 |
| 2 | MCT Only (Vehicle) | BID | 8 | 2 | 8 | 0 | 14 |
| 3 | *NTP42* | 0.25 mg/kg BID | 8 | 0 | 8 | 2 | 14 |
| 4 | Sildenafil | 50 mg/kg BID | 8 | 2 | 6 | 0 | 12 |
| 5 | Selexipag | 1 mg/kg BID | - | - | 6 | 0 | 6 |
| **Animal deaths that occurred following MCT injection and before terminal surgery* | | | | | | | |
| *^#^ Total animals surviving through to terminal surgery* | | | | | | | |

***Supplemental Table 1B***

| **Group**  **#** | **Group Description** | **Treatment Dose** | ***Cohort #3 (N)*** | | **Total^#^** |
| --- | --- | --- | --- | --- | --- |
|  |  |  | **Enrolled** | **Deaths*** |  |
| 1 | No MCT (Vehicle) | QD | 9 | 0 | 9 |
| 2 | MCT Only (Vehicle) | QD | 13 | 5 | 8 |
| 3 | *NTP42* | 0.125 mg/kg QD | 13 | 6 | 7 |

**Animal deaths that occurred following MCT injection and before terminal surgery*

*# Total animals surviving through to terminal surgery*

***Supplemental Table 2***

| **Prostanoid Receptor** | **Cell Line*** | **Assay Type/Detection Method^$^** |
| --- | --- | --- |
| **Prostaglandin D_2_ Receptor 1 (DP1)** | HEK 293 | cAMP/HTRF |
| **Prostaglandin E_2_ Receptor 1 (EP1)** | HEK 293 | [Ca^2+^]_i_/Fluorimetry |
| **Prostaglandin E_2_ Receptor 2 (EP2)** | HEK 293 | cAMP/HTRF |
| **Prostaglandin E_2_ Receptor 3 (EP3)** | HEK 293 | Impedance/Cellular dielectric spectroscopy |
| **Prostaglandin E_2_ Receptor 4 (EP4)** | HEK 293 | cAMP/HTRF |
| **Prostaglandin F_2_ Receptor (FP)** | HEK 293 | [Ca^2+^]_i_/Fluorimetry |
| **Prostaglandin I_2_ Receptor (IP)** | HEK 293 | cAMP/HTRF |
| **Thromboxane A_2_ Receptor α (TPα)** | HEK 293 | [Ca^2+^]_i_/Fluorimetry |
| ******* *Specific* *mammalian cell line* *used typically over-expressed the given receptor.* | | |
| *^$^ Depending on the target GPCR evaluated,* *the assay type and method of detection varied as specified.* | | |

**Supplementary Figures**

***
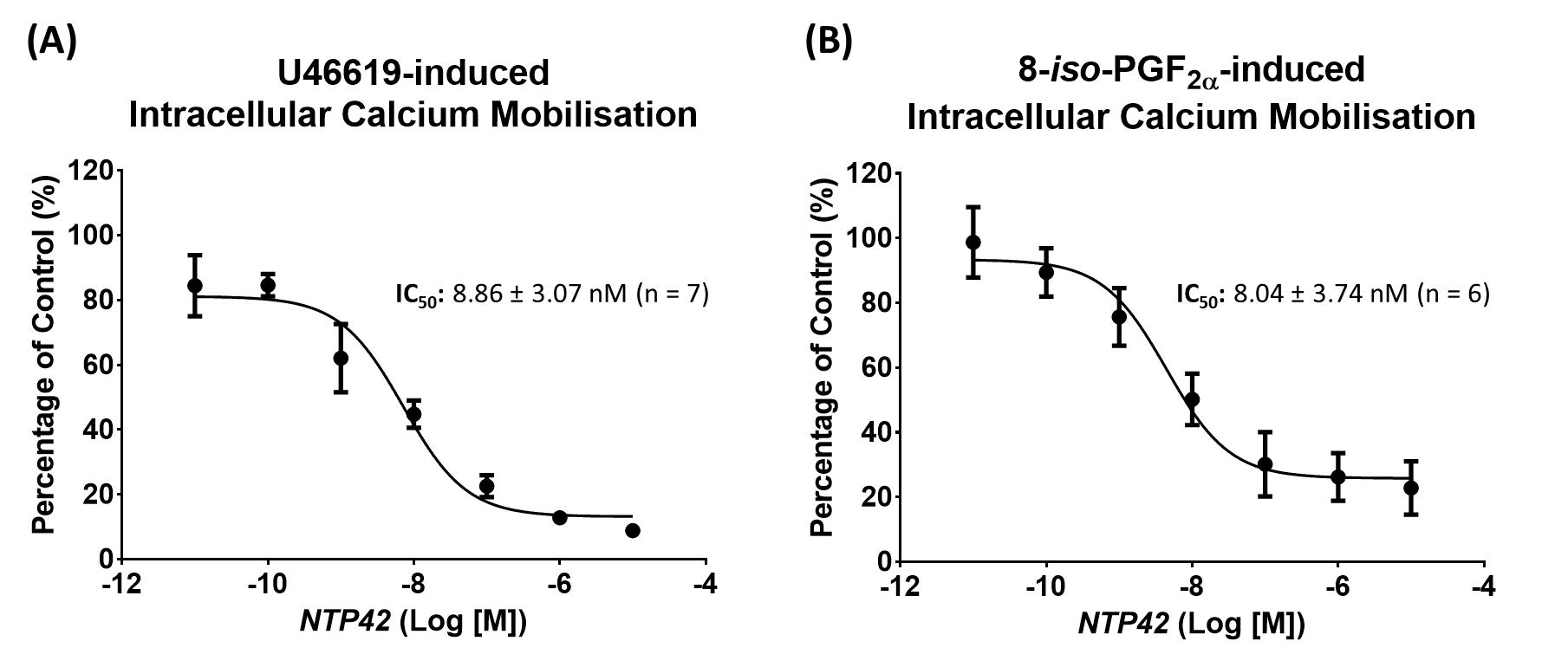
***

***Supplemental Figure 1.* Effect of *NTP42* on intracellular calcium mobilisation following U46619 or 8-iso-PGF_2α_ stimulation.**

HEK.TPβ cells, preloaded with Fluo-4, were incubated with *NTP42* (0.00001 - 10 µM), prior to stimulation with the TP agonists **(A)** 1 μM U46619 or **(B)** 10 μM 8-iso-PGF_2α_. In both panels, dose inhibition curves show the effect of *NTP42* on intracellular Ca^2+^ mobilisation following stimulation with the respective TP agonist. Data is presented as the mean (± SEM) percentage of the respective agonist-induced response in vehicle-treated cells (Percentage of Control; %) and represents data from at least 6 independent experiments (n ≥ 6). Calculated IC_50_ values for *NTP42* are shown alongside the respective dose inhibition curve.

***
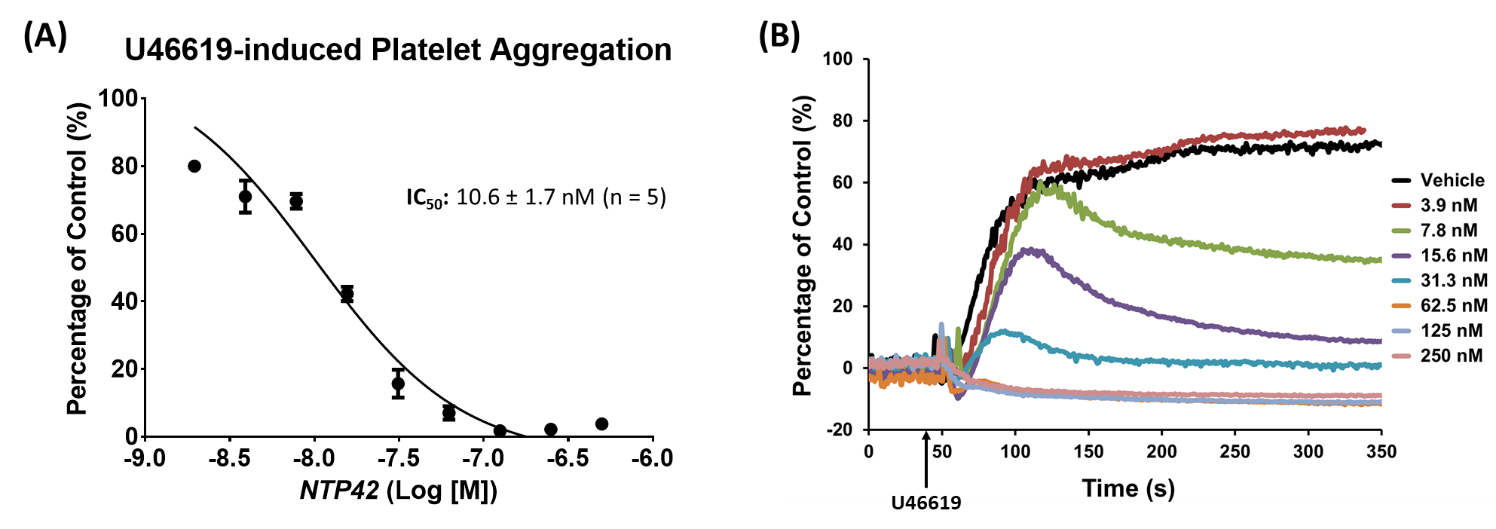
***

***Supplemental Figure 2.* Effect of *NTP42* on human platelet aggregation following U46619 stimulation.**

**(A)** Dose inhibition curve showing the effect of *NTP42* (3.9 - 250 nM) on platelet aggregation following stimulation with 1 μM U46619. Data is presented as the mean (± SEM) changes in percentage aggregation (Percentage of Control; %), as determined by changes in light transmission over time using the PAP-8E Platelet Aggregation Profiler and are representative of at least 7 independent experiments (n = 5). The calculated IC_50_ value for *NTP42* is shown alongside the dose inhibition curve. **(B)** Representative experimental traces showing the effect of *NTP42* (3.9 - 250 nM, as indicated) on platelet aggregation following stimulation with 1 μM U46619.


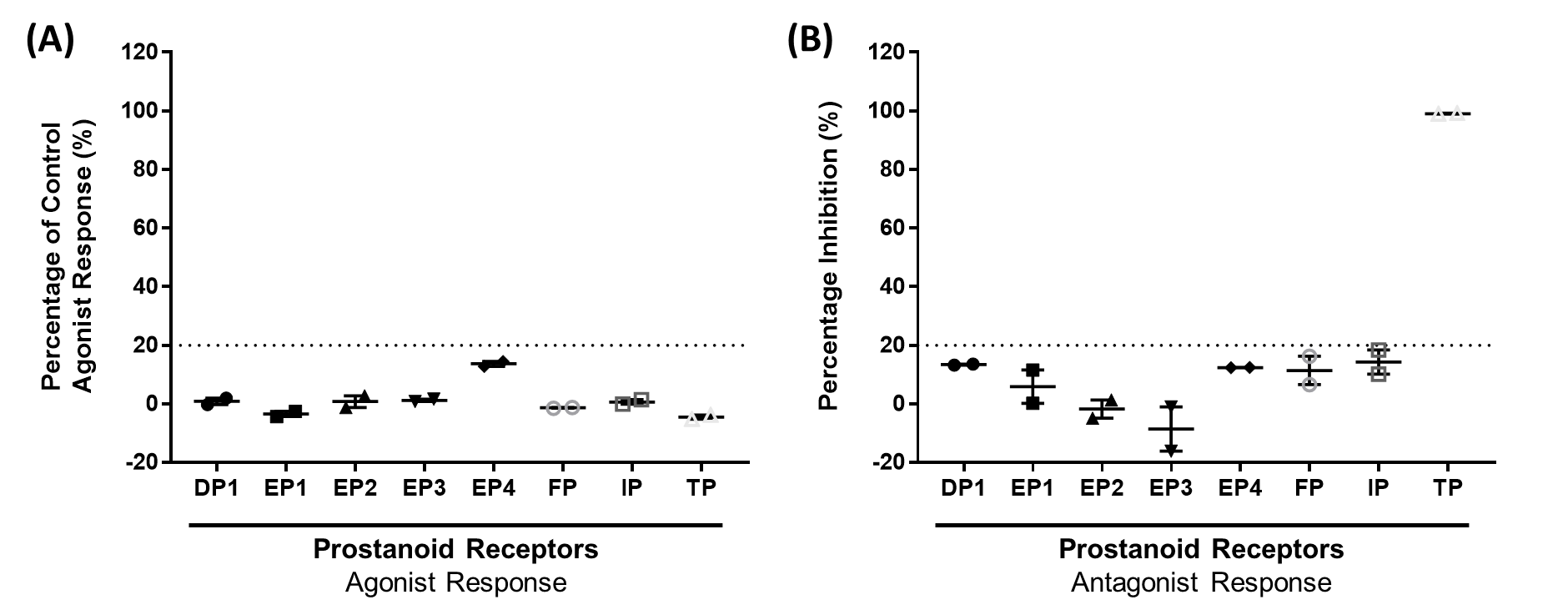


***Supplemental Figure 3.* Specificity of *NTP42* as agonist or antagonist at prostanoid GPCRs.**

*NTP42* (1 µM) was evaluated as an agonist **(A)** or antagonist **(B)** of the TP and 7 other prostanoid receptors, where the mammalian cell line used as source of the given test receptor, along with the assay type and method of detection used is specified in *Supplemental Table 2*. **(A)** As a reference control, the specific cell type was stimulated with a given concentration of a recognised agonist necessary to bring about a maximal response at the given/target receptor (e.g. for the TP with 0.3 μM U46609). Results were calculated and are presented as percentage of the agonist-response following stimulation of cells with *NTP42* (1 µM) relative to the agonist-response following stimulation of cells with the given concentration of the recognised agonist known to bring about a maximal response, set at 100%. **(B)** For assays of receptor antagonism, the specific cell type used for the given receptor was co-stimulated with the test compound (*NTP42*; 1 µM) in the presence of a given concentration of a recognised agonist necessary to bring about a sub-maximal response at the given/target receptor (e.g. for the TP with 10 nM U46609). Results were calculated and are presented as percentage of inhibition of the response generated in the presence of the test drug *NTP42* (1 µM) relative to the agonist-response following stimulation of cells with the given concentration of the recognised agonist known to bring about a sub-maximal response, set at 100%.

***Supplemental Figure 4.* Effect of *NTP42* on Thromboxane A_2_ Synthase Activity.**

*NTP42* (1 µM), the reference compound Furegrelate (10 pM to 3 µM), the drug vehicle (1% DMSO) or water (Control) were preincubated in duplicate for 15 min at 22°C with the thromboxane (TX) A_2_ synthase enzyme preparation from human platelets (15 µg) in a buffer containing 50 mM Tris-HCl (pH 7.5), 70 µg/ml PMSF, 10 µg/ml trypsin inhibitor, 1 µg/ml leupeptin and 1 µM pepstatin. The reaction was initiated by adding 10 µM PGH_2_ and the mixture was incubated for 2 min at room temperature (22°C). For basal control measurements, the enzyme preparation was omitted from the reaction mixture. Following incubation, the reaction was stopped by adding 1 M HCl and the pH neutralised by adding 1 M Tris/HCl at pH 8. The amount of TXB_2_, the stable hydration product of TXA_2_ generated during reaction, was quantified using the EIA (enzyme immunoassay) detection kit (R&D Systems) and the measurements were made with a microplate reader (EnVision, Perkin Elmer). Results were expressed as the percentage of TXB_2_ levels generated in the control reaction, in the absence of an TXA_2_S inhibitor.

***
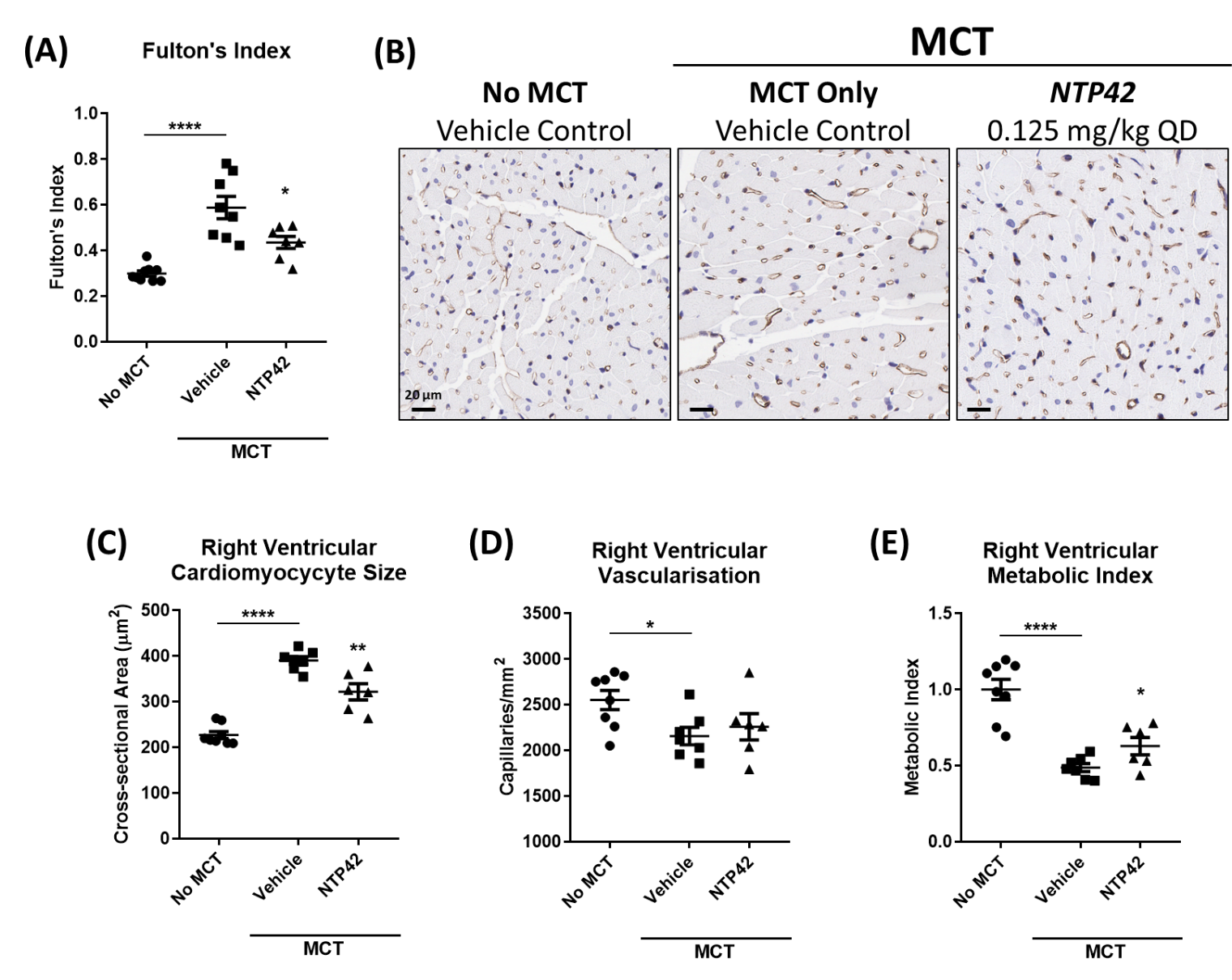
***

***Supplemental Figure 5.* Effect of *NTP42* on cardiac hypertrophy and vascularization.**

Right ventricular hypertrophy-related measurements of: **(A)** Fulton’s Index in the ‘No MCT’, ‘MCT Vehicle’, and *NTP42* groups [n = 9, 8 and 7, respectively]; **(C)** Cardiomyocyte size [n = 8, 7 and 6, respectively]; **(D)** Cardiac vascularisation [n = 8, 7 and 6, respectively] and **(E)** Metabolic Index [n = 8, 7 and 6, respectively], from an additional experimental cohort (Cohort #3, see *Supplemental Table 1B*). All data are expressed as the mean ± SEM. * P ≤ 0.05, ** P ≤ 0.01 and **** P ≤ 0.0001 vs. ‘MCT Vehicle’, according to unpaired Student’s t tests. Panel **(B)** shows representative photomicrographs of *anti-*CD31-stained right ventricle sections from a randomly selected animal from each group. Images shown were captured at 400× magnification. Black scale lines represent 20 μm. Note that while *Supplemental Table* *1B* provides details on numbers of animals enrolled into the study and those that survived through to terminal surgery, the numbers (n) given in the square brackets in the figure legend refer to the number of input data used for the given experimental parameter following removal of any justifiable outliers identified using the method of Interquartile Range (IQR) with Tukey fences.

**Supplementary References**

1. Turner EC, Mulvaney EP, Reid HM, Kinsella BT. Interaction of the Human Prostacyclin Receptor with the PDZ Adapter Protein PDZK1: Role in Endothelial Cell Migration and Angiogenesis. Mol Biol Cell. 2011;22(15):2664-79.

2. Wikstrom K, Kavanagh DJ, Reid HM, Kinsella BT. Differential regulation of RhoA-mediated signaling by the TPalpha and TPbeta isoforms of the human thromboxane A2 receptor: independent modulation of TPalpha signaling by prostacyclin and nitric oxide. Cell Signal. 2008;20(8):1497-512.

3. Bankhead P, Loughrey MB, Fernandez JA, Dombrowski Y, McArt DG, Dunne PD, et al. QuPath: Open source software for digital pathology image analysis. Sci Rep. 2017;7(1):16878.

4. Schermuly RT, Dony E, Ghofrani HA, Pullamsetti S, Savai R, Roth M, et al. Reversal of experimental pulmonary hypertension by PDGF inhibition. J Clin Invest. 2005;115(10):2811-21.

5. Walsh MT, Foley JF, Kinsella BT. The alpha, but not the beta, isoform of the human thromboxane A2 receptor is a target for prostacyclin-mediated desensitization. J Biol Chem. 2000;275(27):20412-23.

6. Kassack MU. Quantitative comparison of functional screening by measuring intracellular Ca2+ with radioligand binding at recombinant human dopamine receptors. AAPS PharmSci. 2002;4(4):E31.
